# Efficacy and safety of TiaoPi AnChang decoction to reduce CAPOX-Induced adverse reactions in Chinese patients with colorectal cancer: a randomized, double-blind, placebo-controlled trial

**DOI:** 10.3389/fphar.2026.1707147

**Published:** 2026-05-29

**Authors:** Jincheng Zhang, Yantong Guo, Zhan Hua, Meng Liu, Tao Fu, Wu Ning, Lei Zhou, Tao Tang, Xin Song, Chao Wang, Shaoxuan Chen, Dakui Zhang, Haibin Liu, Guochao Zhang, Jianzheng Jie, Zhiqiang Cheng

**Affiliations:** 1 Graduate School, Beijing University of Chinese Medicine, Beijing, China; 2 Department of Integrated Traditional Chinese and Western Medicine Oncology, China-Japan Friendship Hospital, Beijing, China; 3 Department of Gastrointestinal Surgery, China-Japan Friendship Hospital, Beijing, China; 4 Department of Colorectal Surgery, China-Japan Friendship Hospital, Beijing, China

**Keywords:** CAPOX-induced adverse reactions, colorectal cancer, randomized controlled trial, TiaoPi AnChang decoction, traditional Chinese medicine

## Abstract

**Purpose:**

This study evaluated the efficacy of TiaoPi AnChang Decoction (TPACD) against CAPOX-induced adverse reactions (CIARs) and improving survival outcomes in colorectal cancer (CRC) patients receiving CAPOX as adjuvant chemotherapy. This paper presents an interim analysis of the clinical trial, with a primary focus on CIARs.

**Patients and methods:**

This was a randomized, double-blind, placebo-controlled trial enrolling high-risk stage II or III CRC patients undergoing CAPOX who were diagnosed with spleen-kidney deficiency syndrome. The study planned to randomly assign 98 patients in a 1:1 ratio to either the TPACD group (30 mL orally, twice daily) or the placebo group (matched placebo). The trial duration spanned 4 chemotherapy cycles (approximately 84 days), during which we assessed the incidence of CIARs (graded via CTCAE), Traditional Chinese Medicine (TCM) syndrome scores, quality of life (QoL, evaluated via EORTC QLQ-C30), peripheral blood immune cell subsets, tumor markers, safety, and survival outcomes. A linear mixed-effects model was applied to adjust for baseline differences.

**Results:**

Among 496 screened CRC patients, 108 were randomized into the TPACD group (n = 54) or the placebo group (n = 54). The incidence of overall CIARs was significantly lower in the TPACD group (*P* = 0.009), particularly for grade ≥2 CIARs (37.77% vs. 67.70%, *P* = 0.008). Subgroup analyses revealed that the treatment effect was homogeneous, with no significant interaction observed between the treatment and the variables of age, sex, BMI, tumor size, or TNM stage (all *P* for interaction >0.05). TPACD administration resulted in improvements in symptoms including fatigue, nausea, vomiting, loss of appetite, and oxaliplatin-induced peripheral neuropathy. However, its efficacy in alleviating myelosuppression, hand-foot syndrome, and pain was limited. Additionally, TPACD improved overall QoL (least squares mean difference (LSMD) −11.71, *P* < 0.001), attenuated the progression of spleen-kidney deficiency syndrome (LSMD 3.09, *P* < 0.001), and positively influenced the CD4+/CD8+ ratio (1.91 ± 0.94 vs. 2.64 ± 1.04, *P* = 0.002). No additional hepatic or renal injury was observed. The predefined endpoints for survival outcomes had not been reached at the time of this analysis.

**Conclusion:**

TPACD serves as an effective, safe, well-tolerated, and readily acceptable complementary therapy for CRC patients undergoing CAPOX.

**Clinical Trial Registration:**

https://www.chictr.org.cn/index.html, identifier ChiCTR2200065759.

## 1 Introduction

Colorectal cancer (CRC) remains a major public health burden in China, ranking as the second most common malignancy and the fourth leading cause of cancer-related mortality (Zheng et al., 2024). Distant metastasis is a critical determinant of the overall survival (OS) of CRC patients. Those without metastasis have a 5-year OS rate approaching 80% following standard treatment (Yothers et al., 2011), whereas it plummets to below 20% in patients with metastatic CRC (Van Cutsem et al., 2011). However, even patients initially presenting with localized tumors remain at risk of developing metastatic disease. The cornerstone of curative treatment is surgical resection of the primary tumor, routinely followed by adjuvant chemotherapy to eradicate occult micrometastases and circulating tumor cells.

The 2024 Chinese Society of Clinical Oncology (CSCO) guidelines for CRC management recommend capecitabine plus oxaliplatin (CAPOX) as a preferred adjuvant chemotherapy regimen for postoperative patients with high-risk stage II and stage III CRC (Wang F. et al., 2025). While CAPOX provides survival benefits comparable to those of 5-fluorouracil plus leucovorin plus oxaliplatin (mFOLFOX6), it offers the advantage of significantly reducing the intravenous infusion time by at least 2 days. Nonetheless, the shift from intravenous 5-fluorouracil to oral capecitabine has not mitigated the burden of CAPOX-induced adverse reactions (CIARs), such as hand-foot syndrome (HFS), oxaliplatin-induced peripheral neuropathy (OIPN), myelosuppression, and CAPOX-induced gastrointestinal toxicities (CIGTs) (André et al., 2018). On the contrary, CAPOX is associated with a significantly increased occurrence of grade ≥3 diarrhea and vomiting, as well as HFS (Yoshino et al., 2022). CIARs profoundly impair patients’ quality of life (QoL) and frequently necessitate dose reductions or treatment discontinuation, which can ultimately compromise treatment efficacy. Although targeted supportive medications have been developed and are extensively utilized to manage these toxicities, their inherent adverse reactions, including fatigue, constipation, loss of appetite, and bone pain, often compound chemotherapy-induced symptoms, further complicating clinical management (Epstein et al., 2020; Hata et al., 2022).

Multiple meta-analyses indicate that Traditional Chinese Medicine (TCM) interventions improve chemotherapy completion rates among CRC patients by mitigating a spectrum of CIARs (Jiang et al., 2023; Han et al., 2024). However, clinical observations suggest that CIGTs, including anorexia, nausea, and vomiting, compromise patient adherence to oral medications (Andreyev et al., 2021). Moreover, the unpalatable odor and taste of traditional Chinese decoctions can often exacerbate these gastrointestinal symptoms. Classical TCM texts, including *Zhouhou BeijiFang* and *Shiliao Bencao*, contain records of numerous medicinal materials with favorable palatability, and these plants, fungi, and zoological materials are currently used in medicines and foods in China. Recognized for their therapeutic efficacy, these materials are listed in the Pharmacopoeia of the People’s Republic of China; concurrently, due to their palatability and nutritional value, they are widely used as foods (Sun et al., 2025). Consequently, combining plants, fungi, and zoological materials used in medicines and foods with adjuvant chemotherapy represents a promising strategy to enhance oral adherence and tolerability in patients with CRC.

TiaoPi AnChang Decoction (TPACD) was developed as an institutional preparation by the China-Japan Friendship Hospital. Derived from from the classical formulation Xiangsha Liujunzi Decoction (XSLJZD), it has been rationally refined to invigorate spleen, tonify kidney, regulate Qi, resolve dampness, harmonize the stomach, and soothe the intestine. XSLJZD has a centuries-long history of clinical application in gastrointestinal oncology (Ni and Ji, 2022), and the efficacy of XSLJZD for CRC has been demonstrated in both clinical and preclinical models (Bao et al., 2021; Chen and Li, 2024). Notably, all constituent medicinal materials of TPACD are formally recognized in China’s official catalog of Substances Recognized as both Food and Medicine. Consequently, TPACD possesses a palatable taste, considerable nutritional value, and a robust safety profile comparable to that of daily dietary foods. TPACD is specifically employed as a complementary therapy during adjuvant chemotherapy for CRC and has been utilized in clinical practice for over 6 years. Previous retrospective cohort studies have demonstrated its potential to mitigate various toxicities associated with CAPOX and to enhance disease-free survival (DFS) rates (He and Cheng, 2023). Mechanistic investigations further indicate that the efficacy of TPACD may be attributable to its bioactive metabolites, including luteolin, quercetin, kaempferol, and liensinine, which exert their effects through modulation of gut microbiota, regulation of microRNA expression, and alteration of C-X-C motif chemokine ligand 8 and matrix metallopeptidase 3 expression levels (Guo et al., 2024; Sun X. et al., 2024; Wang et al., 2024; Wang Y. et al., 2025).

**FIGURE 1:**
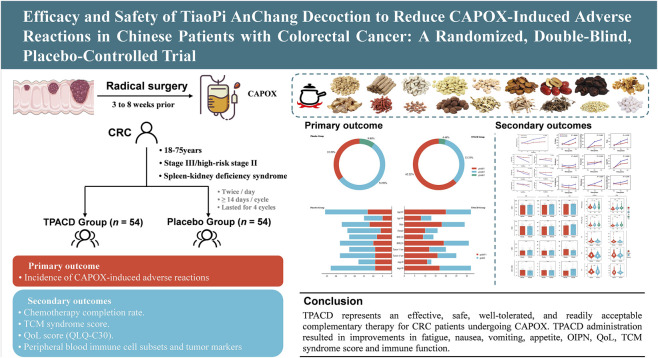
Graphical abstract.

A more rigorously designed clinical trial was initiated to substantiate the efficacy of TPACD in mitigating toxicity and improving DFS and OS when combined with CAPOX. This prospective, randomized, double-blind, placebo-controlled trial was implemented across four departments within China-Japan Friendship Hospital. The study specifically enrolled patients with high-risk stage II or III CRC presenting with spleen-kidney deficiency syndrome who were scheduled to receive adjuvant CAPOX. The trial aimed to evaluate the impact of TPACD on the incidence of CIARs, TCM syndrome scores, QoL, peripheral blood immune cell subsets, tumor markers, and survival outcomes. The co-primary endpoints of this study were the incidence of CIARs, DFS, and OS. Because the survival data had not yet matured at the time of this interim analysis, the current report focuses exclusively on the CIAR endpoint (Figure 1).

## 2 Materials and methods

### 2.1 Study design

This clinical trial utilized a randomized, double-blind, placebo-controlled design and commenced in November 2022, ultimately enrolling 110 participants recruited from four distinct departments within the China-Japan Friendship Hospital. The first participant signed the informed consent form on 5 December 2022, and the final participant completed the study follow-up on 20 December 2024. Random assignment allocated participants in a 1:1 ratio to either the TPACD group or the placebo group for a 12-week treatment duration.

Recruitment sources included the following departments: Department of Integrated Traditional Chinese and Western Medicine Oncology, Colorectal Surgery, Gastrointestinal Surgery, and the Day Ward. The study protocol and all recruitment advertisements received formal approval from the China-Japan Friendship Hospital’s Clinical Research Ethics Committee (2022-KY-114) on 19 July 2022. Furthermore, the trial was prospectively registered with the Chinese Clinical Trial Registry (ChiCTR2200065759) on 14 November 2022.

### 2.2 Participants

In this clinical trial, participant eligibility was determined by attending physicians possessing a minimum of 5 years of experience in integrated traditional Chinese and Western medicine oncology. The inclusion criteria were as follows: (1) A postoperative pathological diagnosis of CRC classified as high-risk stage II (T3N0M0/pMMR with high-risk factors, or T4N0M0) or stage III (any T, N+, M0); (2) Completion of radical CRC surgery 3–8 weeks prior, with satisfactory postoperative recovery, and a planned course of at least 4 cycles of CAPOX; (3) Fulfillment of the diagnostic criteria for TCM spleen-kidney deficiency syndrome, which required the presence of either at least two principal symptoms or one principal symptom concurrent with two secondary symptoms, in addition to characteristic tongue and pulse manifestations. The principal symptoms included 1) sallow complexion; 2) spiritlessness, disinclination to talk, or fatigue and lack of strength; 3) poor appetite and reduced food intake; 4) soreness and weakness in the loins and knees; 5) loose stools. Secondary symptoms included: 1) abdominal distension postprandially; 2) persistent vague abdominal discomfort; 3) clear abundant urine; 4) epigastric fullness; 5) periumbilical pain accompanied by borborygmus and diarrhea, with pain alleviated following defecation; 6) facial or limb edema. Characteristic tongue findings included a pale tongue with a white coating or an enlarged tongue with a white and slippery coating. Pulse presentation typically involved a deep and thin pulse or a deep and weak pulse. (4) Age between 18 and 75 years, and an Eastern Cooperative Oncology Group (ECOG) performance-status score of 0 or 1; (5) Essentially normal function of vital organs or systems, including the heart, brain, liver, lungs, kidneys, endocrine system and hematopoietic system, and the absence of severe medical comorbidities; (6) Willingness and ability to comply with study follow-up procedures and to provide written informed consent.

The exclusion criteria for this study were as follows: (1) Currently or previously having another primary malignant tumor; (2) Having an allergic constitution or being allergic to the any metabolites of the formulation; (3) Having a history of any disease or medication that affects cognitive ability; (4) Being currently involved in or having participated in another clinical trial within the past month; (5) Having undergone neoadjuvant radiotherapy or neoadjuvant chemotherapy or having a concurrent radiotherapy plan; (6) Currently having an active infection; (7) Pregnant women, lactating women, or women in the preconception period.

### 2.3 Sample size estimation

The primary endpoint of this study was the incidence of CIARs. Utilizing relevant data on CIARs from the IDEA trial (André et al., 2018), the incidence of grade ≥3 events was set at 29% for the placebo group in this study. For the TPACD group, this incidence was estimated at 7%, based on previous clinical observational studies (He and Cheng, 2023). Sample size calculation assumed a two-sided alpha (α) of 0.05 for type I error and beta (β) of 0.2 for type II error, with an estimated dropout rate of 10%. A total of 49 patients were needed per group.

### 2.4 Randomization and blinding

A randomization sequence was generated using SPSS (version 27.0; IBM Corp., Armonk, NY, USA). Randomization codes were assigned using a randomized block design with a block size of 4. Drug packaging numbers matched these randomization codes. Eligible subjects were randomly allocated in a 1:1 ratio to either the TPACD group or the placebo group based on the sequence of their baseline assessments. The placebo was indistinguishable from TPACD regarding odor, texture, specification, and appearance. A double-blind design was employed to ensure that participants, investigators, and the statistical team remained blinded to group assignments throughout the enrollment period. The random number table and group allocation list were sealed within opaque envelopes to form the primary blind repository. Primary unblinding occurred after data verification and database locking. During this phase, only group codes (A/B) were disclosed, without revealing the specific interventions assigned. Secondary unblinding was conducted following the completion of the final statistical analysis.

### 2.5 Study medication

#### 2.5.1 Medicinal materials and authentication

TPACD is composed of 18 materials used in medicines and foods, including 16 plants, one fungus, and one zoological material. All botanical materials were validated against World Flora Online (http://www.worldfloraonline.org). The single fungal material was verified using the Index Fungorum database (http://www.indexfungorum.org), while the single zoological material was verified using Chinese Medicine Specimen Database of Hong Kong Baptist University (https://library.hkbu.edu.hk/electronic/libdbs/scm_specimen.html). The TPACD medicinal materials were obtained from Beijing Meikangtang Pharmaceutical Technology Co., Ltd. (Beijing, China), a pharmaceutical manufacturer operating under the supervision and regulation of China’s National Medical Products Administration, and complied with the Pharmacopoeia of the People’s Republic of China (2020 edition). Voucher specimens for Medicinal materials have been deposited in the Clinical Research Center of China-Japan Friendship Hospital (Voucher specimen numbers: 2022-NHLHCRF-TPACD-01–18). The detailed composition of TPACD, including scientific names, families, medicinal parts, harvest locations and seasons, daily dosages, and voucher specimen numbers, is listed in Table 1. Details of the TPACD medicinal materials quality certification are provided in Supplementary Material S1.

**TABLE 1:** Detailed information of TPACD.

Chinese name	Drug name (pharmacopoeial)	Scientific name (Latin)	Family	Medicinal part	Harvesting place	Harvesting season	Daily dose (g)	Voucher specimen number
Huangqi	Astragali radix	*Astragalus mongholicus* Bunge	Fabaceae	Root	Gansu, China	Spring or autumn	15	THQ -01
Dangshen	Codonopsis radix	*Codonopsis pilosula* (Franch.) Nannf	Campanulaceae	Root	Gansu, China	Autumn	10	TDS-02
Fuling	Poria	*Wolfiporia cocos* (F.A. Wolf)Ryvarden and Gilb	Apiaceae	Sclerotium	Anhui, China	Summer (July–September)	10	TFL-03
Yiyiren	Coicis semen	*Coix lacryma*-*jobi* L	Poaceae	Seed	Fujian, China	Autumn	10	TYYR-04
Shanyao	Dioscoreae rhizoma	*Dioscorea acerifolia* Phil	Dioscoreaceae	Rhizome	Henan, China	Winter	10	TSY-05
Lianzi	Nelumbinis semen	*Nelumbo nucifera* Gaertn	Nelumbonaceae	Seed	Hunan, China	Autumn	10	TLZ-06
Sharen	Amomi fructus	*Amomum villosum* Lour	Zingiberaceae	Fruit	Guangdong, China	Summer–Autumn	6	TSR-07
Foshou	Citri sarcodactylis fructus	*Citrus medica* L	Rutaceae	Fruit	Guangdong, China	Autumn	10	TFS-08
Xiangyuan	Citri fructus	*Citrus* × *wilsonii* Tanaka	Rutaceae	Fruit	Sichuan, China	Autumn	10	TXY-09
Machixian	Portulacae herba	*Portulaca oleracea* L	Portulacaceae	Aerial part	Sichuan, China	Summer–Autumn	10	TMCX-10
Baibiandou	Lablab semen album	*Dolichos lablab* L	Fabaceae	Seed	Anhui, China	Autumn-Winter	10	TBBD-11
Shanzha	Crataegi fructus	*Crataegus pinnatifida* Bunge	Rosaceae	Fruit	Shandong, China	Autumn	10	TSZ-12
Gouqizi	Lycii fructus	*Lycium barbarum* L	Solanaceae	Fruit	Ningxia, China	Summer–Autumn	10	TGQZ-13
Huangjing	Polygonati rhizoma	*Polygonatum sibiricum* F. Delaroche	Geraniaceae	Rhizome	Guizhou, China	Spring or autumn	10	THJ-14
Shaji	Hippophae fructus	*Hippophae rhamnoides* L	Elaeagnaceae	Fruit	Sichuan, China	Autumn-Winter	6	TSJ-15
Jineijin	Galli gigerii endothelium corneum	*Gallus gallus domesticus* Brisson	Phasianidae	Inner lining of the gizzard	Henan, China	N/A	10	TJNJ-16
Zhigancao	Glycyrrhizae radix et rhizoma	*Glycyrrhiza uralensis* Fisch	Fabaceae	Root and rhizome	Neimenggu, China	Spring or autumn	6	TZGC-17
Dazao	Jujubae fructus	*Ziziphus jujuba* Mill	Rhamnaceae	Fruit	Shandong, China	Autumn	5	TDZ-18

#### 2.5.2 Preparation and critical parameters

TPACD Preparation Process: The decoctions were prepared according to a standardized protocol and verified by licensed pharmacists at the Pharmacy Department of the China-Japan Friendship Hospital. Briefly, the medicinal materials were weighed according to these proportions and immersed in 700 mL of cold water and soaked at room temperature for 30 min. The mixture was then heated and maintained at a gentle boil for 30 min before the decoction was filtered off. This extraction process was repeated a second time. The filtrates from both decoctions were combined, concentrated to a final volume of 60 mL, and aseptically packaged into individual 30 mL bags, each labeled with a unique drug identification number.

Drug-Extract Ratio (DER) and Dosage: Each dose of TPACD, consisting of 168 g of medicinal materials, was decocted to a final volume of 60 mL, equivalent to a crude drug concentration of 2.8 g/mL.

Placebo Preparation Process: Given the inherent challenges in formulating a placebo for a decoction derived from materials used in medicines and foods, the Pharmacy Department of China-Japan Friendship Hospital developed a matched placebo. This placebo was formulated using 10% low-dose TPACD (Bian et al., 2017; Deng et al., 2025), aspartame, bittering agents, caramel coloring, and tragacanth gum to meticulously match the authentic TPACD in its visual appearance, odor, texture, and taste profile.

#### 2.5.3 Chemical profiling and quality control system

Ultra-performance liquid chromatography ion mobility quadrupole-time-of-flight tandem mass spectrometry (UPLC-IM-QTOF-MS/MS) and ultra-high-performance liquid chromatography-Q Exactive tandem mass spectrometry (UHPLC-QE-MS/MS) were primarily applied to characterize the chemical specificity of TPACD, thereby establishing a robust and orthogonal chemical profile. This dual-platform approach achieves comprehensive orthogonality across three dimensions: (1) mass analyzer orthogonality (Orbitrap vs. TOF detectors); (2) ionization polarity orthogonality (positive vs. negative ESI modes); and (3) fragmentation logic orthogonality (data-dependent acquisition [DDA] on the QE system vs. data-independent acquisition [DIA/MS^E^] on the Q-TOF system). The metabolite characterization was performed by Fang Wei and Guofang Tian at the Center of Pharmaceutical Technology, Tsinghua University. In our prior study, a comprehensive chemical characterization of the formulated product was systematically conducted utilizing UPLC-IM-QTOF-MS/MS (Guo et al., 2024). The chemical fingerprint of the decoction utilized in the present clinical trial demonstrated a high degree of concordance with the reference fingerprint established in the preliminary study. The chromatography and mass spectrometry conditions, detailed results of the fingerprint analyses, and the specific metabolites identified in TPACD via the UPLC-IM-QTOF-MS/MS and UHPLC-QE-MS/MS systems are comprehensively documented in Supplementary Figure S1; Supplementary Table S1. This analytical process was rigorously guided by ConPhyMP checklist (Heinrich et al., 2022), thereby providing robust evidence for the consistent quality of the investigational product.

#### 2.5.4 A priori safety assessment

Prior to the initiation of this trial, an *a priori* assessment regarding the general safety, potential side effects, and interactions of TPACD was comprehensively conducted (He and Cheng, 2023; Guo et al., 2024). Based on our preliminary murine models and retrospective clinical studies, the concomitant administration of TPACD did not induce additional hepatic or renal impairment, hematological toxicity, or neurotoxicity. Furthermore, TPACD did not compromise the favorable effects of the CAPOX regimen on DFS and OS, indicating the absence of adverse TPACD-drug interactions.

### 2.6 Intervention

CAPOX for each patient was administered in accordance with the CSCO guidelines, and the chemotherapy dose was calculated based on the patient’s body surface area (Yuan et al., 2022). Specifically, oxaliplatin at a dose of 130 mg/m^2^ was infused intravenously over 2 h on Day 1. Capecitabine was administered orally at 1000 mg/m^2^ twice daily from Day 1 to Day 14. Cycles were repeated every 21 days for a minimum of 4 cycles. All patients were premedicated intravenously with 0.25 mg of palonosetron hydrochloride and 40 mg of methylprednisolone sodium succinate 30 min before the oxaliplatin infusion. Patients in the TPACD group received 30 mL of TPACD orally twice daily, starting from the first day of CAPOX until the completion of 4 cycles, with the stipulation that TPACD be taken for a minimum of 14 days within each cycle. Patients in the placebo group received a placebo of the same dosage and administration schedule as those in the TPACD group. The concomitant use of any TCM or their formulations was prohibited throughout the trial. Participants who violated this protocol would be considered to have voluntarily withdrawn from the trial. Concomitant medications, including antiemetics, drugs for managing chronic conditions requiring regular administration, and other essential medications approved by the trial investigators (e.g., analgesics or cold remedies), were permitted.

### 2.7 Outcome measures

#### 2.7.1 Primary outcome measures

The primary endpoint was the grade of CIARs. The overall CIARs grade was defined as the highest grade of all CIARs observed during the follow-up period, whereas the category-specific CIARs grade was defined as the highest grade for each specific adverse event during follow-up. Investigators recorded the incidence and severity of CIGTs (including anorexia, nausea, vomiting, diarrhea, constipation, and oral mucositis), myelosuppression (including leukopenia, neutropenia, anemia, and thrombocytopenia) and HFS according to the U.S. Department of Health and Human Services Common Terminology Criteria for Adverse Events (CTCAE, version 5.0). Additionally, the incidence and severity of OIPN were assessed using the oxaliplatin specific toxicity grading standard (Levi classification). Laboratory parameters (including at least white blood cell count, neutrophil count, hemoglobin level, and platelet count) were assessed weekly following oxaliplatin infusion. The highest grade CIARs experienced by the participants were documented in the case report form (CRF) on day 1 following each chemotherapy cycle and were simultaneously updated in the China-Japan Friendship Hospital Integrated Traditional and Western Medicine General Data Platform. A sample CRF is provided in the Supplementary Material for reference.

#### 2.7.2 Secondary outcome measures

- Chemotherapy completion rate.
- TCM syndrome score: Points are assigned and summed according to the severity of each principal and secondary symptom; a higher total score indicates a greater severity of spleen-kidney deficiency. In the Supplementary Material’s CRF, a graded quantitative scale for spleen-kidney deficiency TCM syndrome score is provided for reference. This indicator is assessed at baseline and on day 1 following each chemotherapy cycle.
- QoL score: Assessments across all dimensions are completed using the European Organization for Research and Treatment of Cancer Quality of Life Questionnaire Core 30 (EORTC QLQ-C30), version 3.0. This indicator is evaluated at baseline and on day 1 after the second and fourth chemotherapy cycles. The EORTC QLQ-C30 assesses 15 domains through its 30 items, which are categorized into 5 functional scales (physical, role, emotional, cognitive, and social functioning), 3 symptom scales (fatigue, pain, and nausea/vomiting), a global QoL scale, and 6 single-item symptom or problem measures (dyspnea, insomnia, loss of appetite, constipation, diarrhea, financial difficulties) (Aaronson et al., 1993). Each domain is scored using a standardized algorithm that linearly transforms raw item scores to a 0–100 scale. For functional scales and the global QoL scale, higher scores indicate better levels of functioning or QoL. Conversely, higher scores on symptom scales reflect greater symptom severity or more frequent problems.
- Peripheral blood immune cell subsets and tumor markers: This indicator is measured at baseline and on day 1 following the second and fourth chemotherapy cycles. The assessment includes, but is not limited to, CD3^+^ (%), CD4^+^ (%), CD4+/CD8+ ratio (%), NK (%), CD19^+^ (%), carcinoembryonic antigen (CEA), and carbohydrate antigen 19-9(CA19-9).

#### 2.7.3 Safety assessment

Vital signs and physical examinations were monitored. Peripheral blood hepatic and renal function parameters, including alanine aminotransferase (ALT), aspartate aminotransferase (AST), total bilirubin (TBIL), alkaline phosphatase (ALP), gamma-glutamyl transferase (GGT), and serum creatinine (Scr), were evaluated at baseline and on the first day following each chemotherapy cycle. All adverse events occurring at any time point were documented (Table 2).

**TABLE 2:** Schedule of assessments.

Timepoints	Vx^a^	V0^b^	V1^c^	V2^d^	V3^e^	V4^f^
(Day)	−56 ∼ - 21	0	21	42	63	84
Collection of the medical history						
Written informed consent	X	​	​	​	​	​
Check the inclusion and exclusion criteria	X	X	​	​	​	​
Observation of the therapeutic index						
CIARs, including RBT (WBC,NEUT,Hb,PLT)	X	X	X	X	X	X
TCM symptom score	​	X	X	X	X	X
Peripheral blood immune cell subsets and tumor markers	​	X	​	X	​	X
The EORTC QLQ-C30 score	​	X	​	X	​	X
Chemotherapy completion rate	​	​	​	​	​	X
Safety evaluation						
Vital signs and physical examination	X	X	X	X	X	X
Hepatic function (ALT,AST,TBIL,ALP,GGT)	X	X	X	X	X	X
Renal function (Scr)	X	X	X	X	X	X
Review of AEs/SAEsAE/SAE	X	X	X	X	X	X

^a^ The screening stage.

^b^ The first day of the first chemotherapy cycle.

^c-f^ The first day after the second – fourth chemotherapy cycle.

### 2.8 Statistical analysis

Data analysis was performed using SPSS software. Continuous variables with normal distribution are expressed as mean ± standard deviation (SD), and group comparisons for these variables were conducted using the independent samples t-test. For continuous variables that were skewed, group comparisons were carried out with the nonparametric Mann-Whitney U test. Categorical variables are presented as frequencies or percentages, and group differences were evaluated using the chi-square test, Fisher’s exact test, or the rank-sum test, as appropriate based on data characteristics and application context. Subgroup analysis of the incidence of overall CIARs was performed using logistic regression, with adjustments for age, sex, BMI, tumor size, and TNM stage. Missing data were addressed through censoring. Two-sided tests were employed, and a *P*-value <0.05 was considered statistically significant.

The Full Analysis Set (FAS) included all randomly assigned participants who received the study intervention and was utilized for assessing baseline characteristics and safety. The Per Protocol Set (PPS), consisting of participants who adhered well to the trial protocol, was used to evaluate the therapeutic effect.

A linear mixed-effects model (LMM) employing Restricted Maximum Likelihood estimation was utilized to further analyze changes in TCM syndrome scores and QoL scores. This approach efficiently managed missing continuous data by utilizing all available data points, thereby avoiding listwise deletion or reliance on data imputation. The model adjusted for baseline differences, incorporated individuals as random effects, and specified an unstructured covariance type for repeated measures. It evaluated the main effects of time, the main effects of intervention, their time-by-intervention interaction effect, and the simple effects of treatment at different time points, using the repeated measures data from both groups.

## 3 Results

### 3.1 Participants characteristics

**FIGURE 2:**
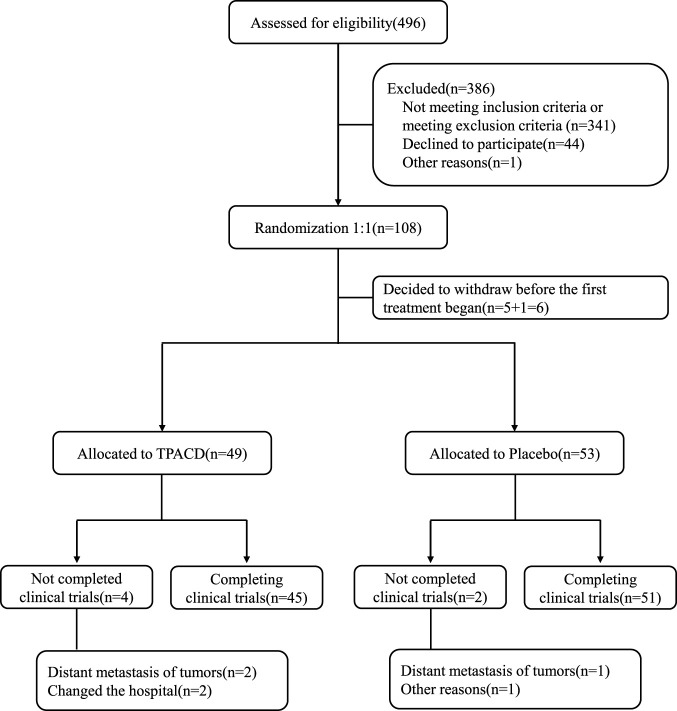
Study flow diagram.

Between 5 December 2022, and 27 September 2024, a total of 496 postoperative patients were screened at the study sites based on broad initial criteria comprising “colorectal cancer” or “postoperative adjuvant chemotherapy for malignant tumor” combined with inpatient oxaliplatin administration to accommodate hospital system constraints. Consequently, to mitigate the risk of an elevated attrition rate associated with the high prevalence of influenza from late 2022 to early 2023, 108 eligible patients were randomized. Among these, 6 patients withdrew prior to the commencement of the first CAPOX cycle, and 6 others discontinued participation due to disease progression or transfer to another hospital. Ultimately, 102 patients were included in the FAS (49 in the TPACD group and 53 in the placebo group), and 96 patients were included in the PPS analysis (45 in the TPACD group and 51 in the placebo group) (Figure 2).

The mean age was 57 years in the TPACD group and 59 years in the placebo group. In the TPACD group, 34% of patients had high-risk stage II disease and 65% had stage III disease, compared to 34% and 66% in the placebo group, respectively. Baseline characteristics were comparable between the two groups, with no statistically significant differences observed (Table 3).

**TABLE 3:** Baseline characteristics (FAS), n (%).

Characteristics	Placebo group (N = 53)	TPACD group (N = 49)	*P*
Age, years	​	​	0.963
<65	37 (69.81)	34 (69.39)	​
≥65	16 (30.19)	15 (30.61)	​
BMI	​	​	0.103
<18.5	0 (0.00)	3 (6.12)	​
>24	24 (45.28)	16 (32.65)	​
18.5 ∼ 23.9	29 (54.72)	30 (61.22)	​
Sex	​	​	0.506
Female	24 (45.28)	19 (38.78)	​
Male	29 (54.72)	30 (61.22)	​
Family tumor history	​	​	0.411
No	50 (94.34)	43 (87.76)	​
Yes	3 (5.66)	6 (12.24)	​
Primary lesion	​	​	0.396
Right colon	11 (20.75)	8 (16.33)	​
Left colon	21 (39.62)	15 (30.61)	​
Rectum	21 (39.62)	26 (53.06)	​
Tumor size> 5 cm	​	​	0.867
No	29 (54.72)	26 (53.06)	​
Yes	24 (45.28)	23 (46.94)	​
Pathological type	​	​	0.126
Containing mucinous adenocarcinoma	5 (9.43)	10 (20.41)	​
Adenocarcinoma	48 (90.57)	38 (77.55)	​
Containing signet ring cell carcinoma	0 (0.00)	1 (2.04)	​
Differentiation	​	​	0.576
Poorly differentiated	3 (5.66)	2 (4.26)	​
Moderately differentiated	39 (73.58)	41 (87.23)	​
Moderately-poorly differentiated	7 (13.21)	3 (6.38)	​
Moderately-well differentiated	3 (5.66)	1 (2.13)	​
Well differentiated	1 (1.89)	0 (0.00)	​
T Staging	​	​	0.456
T1	0 (0.00)	1 (2.04)	​
T2	2 (3.77)	4 (8.16)	​
T3	44 (83.02)	40 (81.63)	​
T4	7 (13.21)	4 (8.16)	​
N staging	​	​	0.727
N0	18 (33.96)	17 (34.69)	​
N1	23 (43.40)	18 (36.73)	​
N2	12 (22.64)	14 (28.57)	​
TNM staging	​	​	0.938
High-risk stage II	18 (34.00)	17 (34.69)	​
Stage III	35 (66.00)	32 (65.30)	​
KRAS	​	​	0.524
Mutant type	14 (27.45)	16 (33.33)	​
Wild type	37 (72.55)	32 (66.67)	​
NRAS	​	​	0.957
Mutant type	1 (1.96)	2 (4.17)	​
Wild type	50 (98.04)	46 (95.83)	​
BRAFV600E	​	​	1
Mutant type	3 (5.88)	2 (4.17)	​
Wild type	48 (94.12)	46 (95.83)	​
Microsatellite	​	​	0.957
MSI-L	1 (1.96)	2 (4.17)	​
MSS	50 (98.04)	46 (95.83)	​

### 3.2 Efficacy

#### 3.2.1 Primary efficacy endpoints

##### 3.2.1.1 The incidence of overall CIARs

The results indicated that all enrolled patients experienced CIARs of varying severity during the follow-up period. The TPACD group demonstrated a significantly reduced overall CIARs grade compared to the placebo group (*P* = 0.009). Furthermore, the majority of CIARs in the TPACD group were classified as grade 1 (62.22% vs. 35.29%, *P* = 0.008) (Supplementary Table S2). Subgroup analyses were conducted based on age, sex, tumor size, and TNM stage. The treatment effect was homogeneous, with no significant interaction observed between the treatment and the variables of subgroup (all *P* for interaction >0.05) (Table 4).

**TABLE 4:** Primary outcome: Incidence of overall CIARs and subgroup analysis (PPS).

Subgroups	N (%)	Placebo group (N = 51)	TPACD group (N = 45)	OR (95%CI)	*P*	*P for interaction*
		*NO. of events(≥2 grade)/NO. of total*				
All patients	96 (100)	33/51	17/45	0.331 (0.144∼0.761)	0.009	​
Age	​	​	​	​	​	0.930
Age ≤65	68 (70.8)	12/32	23/36	0.339 (0.126∼0.910)	0.032	​
Age >65	28 (29.2)	5/13	10/15	0.313 (0.066∼1.472)	0.141	​
Sex	​	​	​	​	​	0.313
Male	56 (58.3)	11/29	15/27	0.489 (0.168∼1.421)	0.189	​
Female	40 (41.7)	6/16	18/24	0.200 (0.051∼0.787)	0.021	​
BMI	​	​	​	​	​	0.850
BMI≤24	59 (61.5)	12/31	18/28	0.351 (0.122∼1.011)	0.052	​
BMI>24	37 (38.5)	5/14	15/23	0.296 (0.074∼1.189)	0.086	​
Tumor	​	​	​	​	​	0.578
Tumor≤5 cm	53 (55.2)	9/25	19/28	0.266 (0.085∼0.832)	0.023	​
Tumor>5 cm	43 (44.8)	8/20	14/23	0.429 (0.126∼1.459)	0.175	​
TNM	​	​	​	​	​	0.567
High-risk stage II	31 (32.3)	2/13	9/18	0.182 (0.031∼1.065)	0.059	​
Stage III	65 (67.7)	15/32	24/33	0.331 (0.118∼0.930)	0.036	​

##### 3.2.1.2 The incidence of CIGTs

Throughout the follow-up period, all patients in both cohorts experienced CIGTs of varying grades. The incidence of overall CIGTs, specifically decreased appetite, nausea, vomiting and diarrhea, differed significantly between the placebo group and the TPACD group (*P* < 0.05). In contrast, the incidence rates of constipation and oral mucositis showed no statistically significant intergroup differences (*P* > 0.05). A significantly greater proportion of patients in the TPACD group experienced overall grade 1 CIGTs compared to the placebo group (93.33% vs. 54.90%, *P* < 0.001). The TPACD group also demonstrated a significantly higher rate of experiencing no loss of appetite (15.56% vs. 0%, *P* = 0.011) and a significantly lower incidence of grade 2 loss of appetite (2.22% vs. 21.57%, *P* = 0.04). Similarly, the absence of nausea was significantly more common (26.67% vs. 1.96%, *P* < 0.001), and grade 2 nausea was significantly less frequent (2.22% vs. 19.61%, *P* = 0.008) in the TPACD group. The TPACD group also showed a markedly higher rate of experiencing no vomiting (84.44% vs. 21.57%, *P* < 0.001). Concurrently, the incidences of both grade 1 (13.33% vs. 58.82%, *P* < 0.001) and grade 2 vomiting (2.22% vs. 17.65%, *P* = 0.033) were significantly lower in the TPACD group. Furthermore, the absence of diarrhea occurred at a significantly higher rate in the TPACD group (71.11% vs. 43.14%, *P* = 0.006), while grade 1 diarrhea was significantly less frequent (26.67% vs. 49.02%, *P* = 0.025) (Table 5). Detailed differences of each grade are available in Supplementary Table S3.

**TABLE 5:** Primary outcome: Incidence of CIGTs (PPS), n (%).

Adverse reactions	Placebo group (N = 51)	TPACD group (N = 45)	*P*
Overall CIGTs	​	​	<0.001
1 grade	28 (54.90)	42 (93.33)	​
2 grade	19 (37.25)	3 (6.67)	​
3 grade	4 (7.84)	0 (0.00)	​
Loss of appetite	​	​	<0.001
0	0 (0.00)	7 (15.56)	​
1 grade	38 (74.51)	37 (82.22)	​
2 grade	11 (21.57)	1 (2.22)	​
3 grade	2 (3.92)	0 (0.00)	​
Nausea	​	​	<0.001
0	1 (1.96)	12 (26.67)	​
1 grade	37 (72.55)	32 (71.11)	​
2 grade	10 (19.61)	1 (2.22)	​
3 grade	3 (5.88)	0 (0.00)	​
Vomiting	​	​	<0.001
0	11 (21.57)	38 (84.44)	​
1 grade	30 (58.82)	6 (13.33)	​
2 grade	9 (17.65)	1 (2.22)	​
3 grade	1 (1.96)	0 (0.00)	​
Diarrhea	​	​	0.005
0	22 (43.14)	32 (71.11)	​
1 grade	25 (49.02)	12 (26.67)	​
2 grade	4 (7.84)	1 (2.22)	​
Constipation	​	​	0.081
0	40 (78.43)	41 (91.11)	​
1 grade	9 (17.65)	4 (8.89)	​
2 grade	2 (3.92)	0 (0.00)	​
Oral mucositis	​	​	0.615
0	35 (68.63)	33 (73.33)	​
1 grade	16 (31.37)	12 (26.67)	​

##### 3.2.1.3 The incidence of myelosuppression

Numerically lower rates was noted in the TPACD group compared to the placebo group for overall myelosuppression (33.33% vs. 42.22%), leukopenia (72.55% vs. 80.00%), neutropenia (64.71% vs. 75.56%), and anemia (72.55% vs. 73.33%), although this difference did not reach statistical significance (Table 6). Detailed differences of each grade are available in Supplementary Table S4.

**TABLE 6:** Primary outcome: Incidence of myelosuppression (PPS), n (%).

Adverse reactions	Placebo group (N = 51)	TPACD group (N = 45)	*P*
Overall myelosuppression	​	​	0.49
0	17 (33.33)	19 (42.22)	​
1 grade	20 (39.22)	15 (33.33)	​
2 grade	13 (25.49)	9 (20.00)	​
3 grade	1 (1.96)	2 (4.44)	​
Leukopenia	​	​	0.368
0	37 (72.55)	36 (80.00)	​
1 grade	8 (15.69)	6 (13.33)	​
2 grade	6 (11.76)	3 (6.67)	​
Neutropenia	​	​	0.17
0	33 (64.71)	34 (75.56)	​
1 grade	7 (13.73)	7 (15.56)	​
2 grade	10 (19.61)	4 (8.89)	​
3 grade	1 (1.96)	0 (0.00)	​
Thrombocytopenia	​	​	0.568
0	38 (74.51)	32 (71.11)	​
1 grade	11 (21.57)	8 (17.78)	​
2 grade	2 (3.92)	3 (6.67)	​
3 grade	0 (0.00)	2 (4.44)	​
Anemia	​	​	0.94
0	37 (72.55)	33 (73.33)	​
1 grade	11 (21.57)	7 (15.56)	​
2 grade	3 (5.88)	5 (11.11)	​

##### 3.2.1.4 The incidence of OIPN and HFS

During follow-up, a statistically significant difference was observed in the incidence of OIPN between the two groups (*P* = 0.003). The TPACD group demonstrated a significantly higher probability of remaining free of OIPN compared to the placebo group (42.22% vs. 11.76%, *P* < 0.001). However, the incidence of grade ≥2 OIPN did not differ significantly between the groups. Although the overall incidence of HFS did not differ statistically between groups during follow-up (*P* = 0.05), the TPACD group had a significantly higher probability of experiencing no HFS compared to the placebo group (75.56% vs. 56.86%, *P* = 0.035). No case of grade ≥3 HFS occurred in either group. The incidence of both OIPN and HFS was associated with Relative Dose Intensity (RDI). A predefined subgroup analysis using an RDI threshold of 85% revealed that a higher RDI was associated with an increased incidence of grade ≥1 OIPN and HFS. In the oxaliplatin subgroups, regardless of whether the RDI was ≥85% or <85%, the proportion of patients developing grade ≥1 OIPN was significantly lower in the TPACD group than in the placebo group (65.2% vs. 92.3%, *P* = 0.046 and 50.0% vs. 84.0%, *P* = 0.013, respectively). In the capecitabine subgroups, regardless of RDI level, no significant difference in HFS incidence was found between the TPACD and placebo groups. Nevertheless, a consistent trend towards a reduction in the number of patients with grade ≥1 HFS was observed in both subgroups (26.1% vs. 48.0%, *P* = 0.117 and 22.7% vs. 38.5%, *P* = 0.241, respectively) (Table 7). Detailed differences of each grade are available in Supplementary Table S5.

**TABLE 7:** Incidence of OIPN and HFS (PPS), n (%).

Adverse reactions	Grades	Placebo group (N = 51)	TPACD group (N = 45)	*P*
OIPN	Total	​	​	0.003
	0	6 (11.76)	19 (42.22)	​
	1 grade	39 (76.47)	22 (48.89)	​
	2 grade	5 (9.80)	4 (8.89)	​
	3 grade	1 (1.96)	0 (0.00)	​
	RDI of oxaliplatin ≥ 85	​	​	0.046
	0	2 (7.7)	8 (34.8)	​
	1 grade	21 (80.8)	13 (56.5)	​
	2 grade	3 (11.5)	2 (8.7)	​
	3 grade	0 (0)	0 (0)	​
	RDI of oxaliplatin < 85	​	​	0.013
	0	4 (16.0)	11 (50.0)	​
	1 grade	18 (72.0)	9 (40.9)	​
	2 grade	2 (8.0)	2 (9.0)	​
	3 grade	1 (4.0)	0 (0)	​
HFS	Total	​	​	0.050
	0	29 (56.86)	34 (75.56)	​
	1 grade	21 (41.18)	11 (24.44)	​
	2 rade	1 (1.96)	0 (0.00)	​
	RDI of capecitabine ≥ 85	​	​	0.117
	0	13 (52.0)	17 (73.9)	​
	1 grade	11 (44.0)	6 (26.1)	​
	2 rade	1 (4.0)	0 (0)	​
	RDI of capecitabine < 85	​	​	0.241
	0	16 (61.5)	17 (77.3)	​
	1 grade	10 (38.5)	5 (22.7)	​
	2 rade	0 (0)	0 (0)	​

#### 3.2.2 Secondary efficacy endpoints

##### 3.2.2.1 Completion rate of planned chemotherapy cycles

Following the exclusion of 6 patients who discontinued the study due to disease progression or changes in treatment centers, the chemotherapy completion rate was 96.08% in the placebo group and 100% in the TPACD group; this difference was not statistically significant (*P* = 0.497). Subgroup analyses of RDI for oxaliplatin and capecitabine demonstrated that, although statistically non-significant, a higher proportion of patients in the TPACD group maintained a capecitabine RDI greater than 85% compared to the placebo group (51.1% vs. 49.0%, *P* = 0.838) (Table 8).

**TABLE 8:** Secondary outcome: Completion rate of chemotherapy (PPS), n (%).

Items	Status	Placebo group (N = 51)	TPACD group (N = 45)	*P*
Completion rate	​	​	​	0.497
	Complete	49 (96.08)	45 (100.00)	​
	Incomplete	2 (0.02)	45 (100.00)	​
Oxalipatin RDI	​	​	​	0.990
	≥85%	26 (51.0)	23 (51.1)	​
	<85%	25 (49.0)	22 (48.9)	​
Capecitabine RDI	​	​	​	0.838
	≥85%	25 (49.0)	23 (51.1)	​
	<85%	26 (51.0)	22 (48.9)	​

##### 3.2.2.2 TCM syndrome score

**FIGURE 3:**
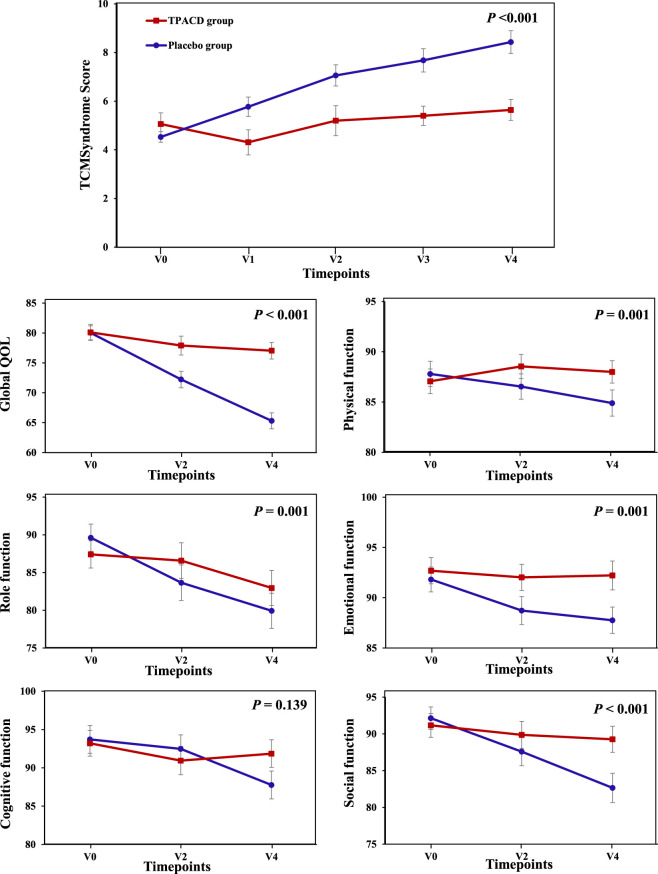
Changes in TCM syndrome score and EORTC QLQ-C30 score across timepoints (PPS), MD ± Standard Error (SE). **(A)** TCM syndrome score. **(B)** Global QoL and functioning scores.

Results from the LMM indicated that both the intervention effect and the time effect significantly influenced the TCM syndrome scores, with a statistically significant intervention-time interaction effect (*P* < 0.001). TCM syndrome scores exhibited an increasing trend over time in both groups, suggesting a progressive worsening of the spleen-kidney deficiency syndrome throughout chemotherapy. Multiple comparisons derived from the LMM at various time points revealed statistically significant differences in TCM syndrome scores between the placebo and TPACD groups at every assessment. The most substantial inter-group difference was noted at time point V4 (least squares mean difference [LSMD] 3.09, 95% confidence interval [CI]: 2.48 to 3.70, *P* < 0.001) (Table 9). In the TPACD group, the severity of the spleen-kidney deficiency syndrome exhibited a transient decrease after the first chemotherapy cycle, followed by a subsequent increase that was less pronounced than that in the Placebo Group. This finding indicates that TPACD exerted a beneficial effect by mitigating the progression of the spleen-kidney deficiency syndrome (Figure 3). Detailed TCM syndrome scores are available in Supplementary Table S6.

**TABLE 9:** Secondary efficacy: TCM syndrome score and QoL score based on LMM (PPS).

Items	Intervention effect			Time effect			Intervention-time interaction effect			V2			V4		
	Est^a^	95%CI	*P*	Est	95%CI	*P*	Est	95%CI	*P*	LSMD^b^	95%CI	*P*	LSMD^c^	95%CI	*P*
TCM syndrome score	2.389	[1.910; 2.868]	<0.001	−2.093	[-2.406; −1.782]	<0.001	−1.29	[-1.900; −0.680]	<0.001	2.19	[1.58, 2.79]	<0.001	3.09	[2.48, 3.70]	<0.001
Global QoL	−8.499	[-11.207; −5.792]	<0.001	4.156	[2.624; 5.688]	<0.001	6.279	[3.474; 9.084]	<0.001	−5.43	[-8.44; −2.41]	0.001	−11.71	[-14.75; −8.66]	<0.001
Physical function	−2.779	[-4.349; −1.208]	<0.001	1.268	[0.516; 2.020]	0.001	0.77	[-0.737; 2.077]	0.313	−2.40	[-4.13; −0.68]	0.007	−3.17	[-4.92; −1.43]	<0.001
Role function	−6.121	[-9.834; −2.409]	0.001	3.934	[1.981; 5.888]	<0.001	2.572	[-1.325; 6.470]	0.193	−4.87	[-9.02; −0.72]	0.022	−7.44	[-11.64; −3.24]	0.001
Emotional function	−3.479	[-5.589; −1.370]	0.001	0.496	[-0.464; 1.455]	0.307	1.302	[-0.609; 3.213]	0.179	−2.85	[-5.14; −0.55]	0.016	−4.15	[-6.47; −1.83]	0.001
Cognitive function	−2.240	[-5.218; 0.739]	0.139	2.315	[0.696; 3.933]	0.006	5.873	[2.843; 8.903]	<0.001	0.63	[-2.68; 3.94]	0.706	−5.24	[-8.58; −1.90]	0.002
Social function	−6.183	[-9.281; −3.085]	<0.001	3.130	[1.063; 5.197]	0.003	5.935	[1.964; 9.906]	0.004	−329	[-6.93; 0.35]	0.076	−9.22	[-12.91; −5.53]	<0.001
Fatigue	7.722	[4.258; 11.185]	<0.001	−5.101	[-6.882; −3.320]	<0.001	−0.338	[-3.922; 3.246]	<0.001	7.56	[3.69; 11.42]	<0.001	7.89	[3.99; 11.80]	<0.001
Nausea and vomiting	15.728	[9.641; 21.813]	<0.001	−3.514	[-6.137; −0.891]	0.009	−5.940	[-11.084; −0.797]	0.024	12.83	[6.28; 19.38]	<0.001	18.77	[12.17; 25.37]	<0.001
Pain	4.496	[-0.512; 9.505]	0.078	−2.907	[-5.225; −0.591]	0.014	−1.259	[-5.917; 3.399	0.593	3.88	[-1.60; 9.36]	0.163	5.14	[-0.38; 10.67]	0.068
Dyspnoea	1.010	[-1.463; 3.485]	0.419	−1.064	[-2.618; 0.489]	0.177	−0.631	[-3.755; 2.493]	0.689	0.70	[-2.19; 3.60]	0.632	1.33	[-1.60; 4.26]	0.37
Insomnia	2.525	[-1.653; 6.703]	0.233	−2.488	[-4.763; −0.213]	0.032	−3.315	[-7.843; 1.214]	0.149	0.91	[-3.79; 5.62]	0.702	4.23	[-0.53; 8.99]	0.081
Appetite	11.428	[3.739; 19.118]	0.004	−5.700	[-9.105; −2.294]	0.001	−2.620	[-9.457; 4.217]	0.449	10.15	[1.80; 18.50]	0.018	12.77	[4.36; 21.19]	0.003
Constipation	2.520	[-1.019; 6.060]	0.161	−0.481	[-2.838; 1.876]	0.686	−2.305	[-7.031; 2.422]	0.335	1.39	[-2.82; 5.60]	0.515	3.70	[-0.56; 7.95]	0.088
Diarrhoea	5.804	[0.414; 11.194]	0.035	−1.538	[-4.701; 1.626]	0.337	−5.694	[-11.959; 0.571	0.074	3.02	[-3.14; 9.19]	0.335	8.72	[2.48; 14.95]	0.006
Financial difficulties	2.973	[-0.242; 6.187]	0.070	−2.502	[-4.545; −0.459]	0.017	−1.927	[-6.021; 2.166]	0.352	2.03	[-1.74; 5.80]	0.289	3.96	[0.14; 7.77]	0.042

a Est., parameter estimates by LMM.

b Adjusted baseline using LMM.

c A positive LSMD, value indicates that the mean score in the TPACD, group was lower than that in the placebo group.

##### 3.2.2.3 QoL by EORTC QLQ-C30

LMM analysis revealed significant main effects for both intervention and time on the Global QoL score, alongside a statistically significant intervention-time interaction effect (*P* < 0.001). Although Global QoL scores demonstrated a declining trend over time in both groups, the TPACD group showed a significantly attenuated decline compared to the placebo group. The most substantial between-group difference was observed at the V4 time point (LSMD -11.71, 95% CI -14.75 to −8.66; *P* < 0.001).

Regarding the 5 functional domains, LMM analysis indicated a significant intervention effect on scores for physical functioning (*P* < 0.001), role functioning (*P* = 0.001), emotional functioning (*P* = 0.001), and social functioning (*P* < 0.001). A significant main effect of time was also observed for physical functioning (*P* = 0.001), role functioning (*P* < 0.001), cognitive functioning (*P* = 0.006), and social functioning (*P* = 0.003). A statistically significant intervention-time interaction effect was identified for the cognitive functioning (*P* < 0.001) and social functioning (*P* = 0.004) domains. Post hoc multiple comparisons revealed that the greatest differences occurred at V4, with the TPACD group showing better outcomes: physical functioning (LSMD -3.17, 95% CI -4.92 to −1.43; *P* < 0.001), role functioning (LSMD -7.44, 95% CI -11.64 to −3.24; *P* = 0.001), emotional functioning (LSMD -4.15, 95% CI -6.47 to −1.83; *P* = 0.001), cognitive functioning (LSMD -5.24, 95% CI -8.58 to −1.90; *P* = 0.002), and social functioning (LSMD -9.22, 95% CI -12.91 to −5.53; *P* < 0.001) (Figure 3).

Analysis of the three symptom domains revealed a significant intervention effect on fatigue (*P* < 0.001) and nausea and vomiting (*P* < 0.001). A significant main effect of time was also noted for fatigue (*P* < 0.001), nausea and vomiting (*P* = 0.009), and pain (*P* = 0.014). Significant intervention-time interaction effects were found for fatigue (*P* < 0.001) and nausea/vomiting (*P* = 0.024). Post hoc analysis indicated no significant between-group difference for pain at any time point. However, for fatigue and nausea/vomiting, differences were greatest at V4, with the TPACD group exhibiting lower symptom scores (Fatigue: LSMD 7.89, 95% CI 3.99 to 11.80; *P* < 0.001; Nausea and vomiting: LSMD 18.77, 95% CI 12.17 to 25.37; *P* < 0.001).

**FIGURE 4:**
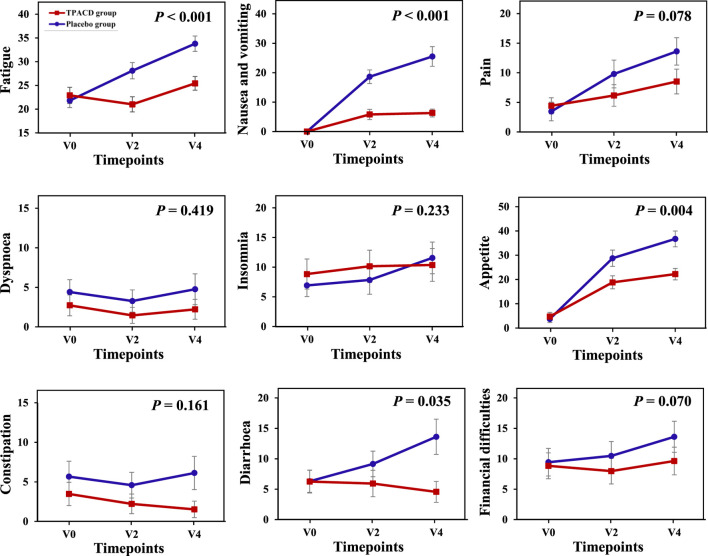
Changes in symptom and single items scores from EORTC QLQ-C30 across timepoints (PPS), Mean ± SE.

For the 6 single items, LMM analysis showed a significant intervention effect on loss of appetite (*P* = 0.004) and diarrhoea (*P* = 0.035). A significant main effect of time was observed for insomnia (*P* = 0.032), appetite loss (*P* = 0.001), and financial difficulties (*P* = 0.017). No significant intervention-time interaction effects were detected for these items (*P* > 0.05). Post hoc tests revealed no significant between-group differences for dyspnoea, insomnia, or constipation. Differences were greatest at V4: appetite loss (LSMD 12.77, 95% CI 4.36 to 21.29; *P* = 0.003), diarrhoea (LSMD 8.72, 95% CI 2.48 to 14.95; *P* = 0.006), and financial difficulties (LSMD 3.96, 95% CI 0.14 to 7.77; *P* = 0.042), with the TPACD group showing better outcomes for appetite loss and diarrhoea (lower scores), and with reduced financial burdens (Figure 4).

Collectively, these findings suggest that TPACD confers a beneficial effect on QoL within the studied population. This benefit is potentially mediated through the amelioration of CIARs, including negative emotional states, somatic symptoms, and impairments in role functioning. Notably, TPACD appears specifically effective in mitigating common CIARs, such as fatigue, nausea, vomiting, appetite loss, and diarrhoea. Detailed data regarding the intervention effect, time effect, intervention-time interaction effect and multiple comparisons at fixed time points calculated by LMM are presented in Table 8. Detailed EORTC QLQ-C30 scores of each domain are available in Supplementary Table S6.

##### 3.2.2.4 Peripheral blood immune cell subsets and tumor markers

**FIGURE 5:**
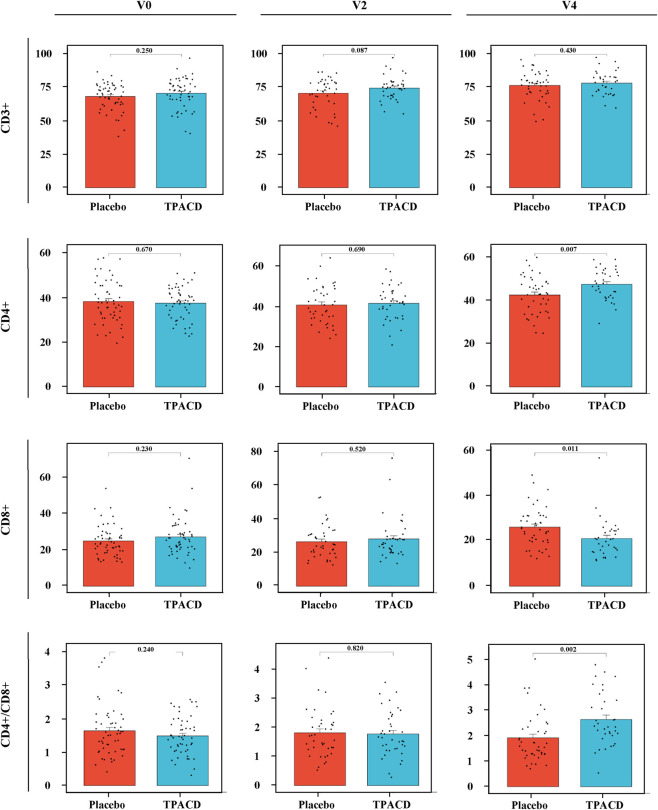
Changes in peripheral blood immune cell subsets across timepoints (PPS), Mean ± SD.

After the fourth cycle of chemotherapy, statistically significant differences were observed in CD4^+^ (%), CD8^+^ (%), and CD4+/CD8+ ratio between the TPACD group and the placebo group. The TPACD group exhibited a higher CD4^+^ percentage (47.68 ± 7.07 vs. 42.73 ± 8.76, *P* = 0.008), a lower CD8^+^ percentage (20.68 ± 8.28 vs. 25.60 ± 8.52, *P* = 0.012), and an elevated CD4+/CD8+ ratio (1.91 ± 0.94 vs. 2.64 ± 1.04, *P* = 0.002) compared to the placebo group (Supplementary Table S7). No significant intergroup differences were detected in CD3^+^ (%), NK (%), or CD19^+^ (%) at any time point. These findings suggest that TPACD may help modulate the balance of peripheral blood lymphocyte subsets (Figure 5).

**FIGURE 6:**
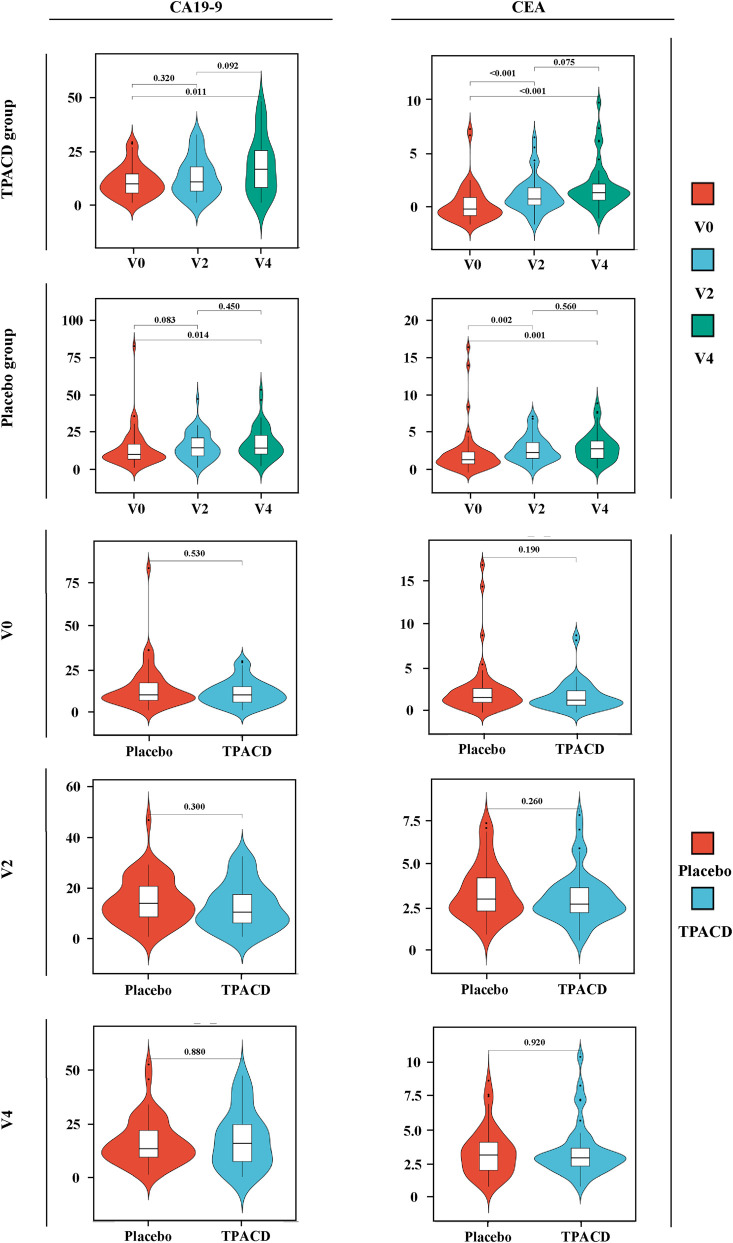
Changes in tumor markers across timepoints (PPS), Median (Lower quartile, Upper quartile).

No statistically significant intergroup differences (*P* > 0.05) were found for CEA or CA19-9 levels at any time points (Supplementary Table S8). Further analysis showed marked increases from baseline in both groups after cycle 4 for CEA (placebo: 1.83 (1.32, 2.80) to 3.14 (1.98, 4.11), *P* = 0.001; TPACD: 1.58 (1.03, 2.56) to 2.93 (2.28, 3.82), *P* < 0.001) and CA19-9 (placebo: 9.87 (6.54, 16.95) to 14.12 (9.94, 23.15), *P* = 0.014; TPACD: 9.84 (5.53, 15.18) to 16.65 (7.93, 25.51), *P* = 0.011). Values, however, remained within normal limits (Supplementary Table S9). The clinical implications of these tumor marker changes require further investigation and discussion (Figure 6).

#### 3.2.3 Safety

Owing to the overlap between many adverse events and adverse reactions associated with CAPOX, this study concentrated on quantifiable myelotoxicity, nephrotoxicity, and hepatotoxicity. Regarding myelotoxicity, as previously described, TPACD did not exacerbate CAPOX-induced myelotoxicity and exhibited a potential ameliorative trend, albeit without statistical significance. Throughout the follow-up period, neither group exhibited renal impairment. The incidence of hepatic injury did not differ significantly between the two groups (49.06% vs. 57.14%, *P* = 0.38). These findings suggest that TPACD is safe for patients receiving CAPOX chemotherapy (Supplementary Table S10).

## 4 Discussion

This randomized, double-blind, placebo-controlled trial evaluated the efficacy of TPACD in high-risk stage II or III CRC patients receiving CAPOX chemotherapy and presenting with spleen-kidney deficiency syndrome. The findings initially affirmed the safety profile of TPACD, indicating no new incidences of hepatic or renal toxicity. Compared to placebo, TPACD reduced the severity of CIARs. Specifically, TPACD administration alleviated CIARs, including fatigue, nausea, vomiting, loss of appetite, and OIPN, and these observations are largely consistent with previous research on analogous TCM formulations for CRC patients experiencing CIARs (Sun L. et al., 2024; Wu et al., 2024). Furthermore, TPACD enhanced overall QoL, notably in the domains of physical and emotional functioning. It also attenuated the progression of spleen-kidney deficiency syndrome and showed potential for regulating subsets of peripheral blood lymphocytes. Conversely, TPACD exhibited limited efficacy in alleviating myelosuppression, HFS, and pain, thereby delineating the boundaries of its therapeutic scope.

Initially, chemotherapy served as a palliative measure for metastatic malignancies before its adoption as postoperative adjuvant therapy aimed at eliminating occult micrometastases to mitigate recurrence risk. Beginning in the 1950s, various agents, including the chemotherapeutic drugs levamisole, fluorouracil, oxaliplatin, irinotecan, and capecitabine, alongside targeted therapies like cetuximab and bevacizumab, were investigated within adjuvant strategies for CRC (Gelibter et al., 2019). Clinical trials NSABP C-08 and NO16968 were pivotal in establishing the mFOLFOX6 and CAPOX, respectively, for adjuvant CRC treatment (Allegra et al., 2011; Schmoll et al., 2015). Nonetheless, significant toxicities associated with these regimens often compromised treatment completion and patient quality of life, which spurred research into shortening the treatment duration. Subsequent IDEA studies and re-analyses of the IDEA study confirmed the survival benefits of the 3-month CAPOX in patients with low-risk stage III (T1-3N1) and high-risk stage II CRC, while also correlating with improved treatment completion, diminished toxicity, and reduced economic costs (Grothey et al., 2018; Iveson et al., 2021). This evidence base directly contributed to the revised 2022 CSCO guidelines for CRC. Consequently, a minimum of 4 cycles (3 months) of adjuvant CAPOX chemotherapy is indicated. Guided by these clinical standards, our study prospectively defined the completion of the fourth CAPOX cycle as the primary milestone for clinical observation and intervention. The chemotherapy completion rate did not differ significantly between the TPACD group and the placebo group (100% vs. 96.08%). These high completion rates are congruent with the 94% adherence reported in the IDEA study, thereby corroborating the favorable tolerability of the 3-month CAPOX.

Nearly 30% of eligible patients declined to participate in this study. Preliminary interviews indicated that over 80% of these patients perceived an increased risk of hepatotoxicity and nephrotoxicity with the concomitant use of TCM during chemotherapy, while more than 50% anticipated difficulty in adhering to the regular intake of TCM decoctions as mandated by the trial protocol, particularly if experiencing nausea and vomiting, even after receiving counseling on potential adverse effects. These concerns highlight the importance of the safety, odor, and taste of TCM. Materials used in medicines and foods address these issues effectively due to their well-recognized safety profile and palatability. Additionally, they offer economic advantages by being more affordable, thereby helping to alleviate the financial burden on patients. Furthermore, they serve as a source of both essential and non-essential nutrients (Xiao et al., 2021). This is particularly relevant given that severe malnutrition is highly prevalent among CRC patients receiving chemotherapy (Attar et al., 2012). Oral nutritional supplementation has been shown to enhance tolerance to chemotherapy and to reduce treatment-related cancer cachexia and treatment interruptions (Lin et al., 2017). Nevertheless, it is noteworthy that the potential for toxicity from overdosing on materials used in medicines and foods remains a non-negligible risk. For example, pharmacological analysis of 7 such materials indicates that their influence on drug-metabolizing enzymes must be considered in the context of long-term use (Zhang et al., 2022).

During chemotherapy, the severity of spleen-kidney deficiency syndrome often worsens, considerably impair QoL, especially in patients who have undergone an ostomy. TPACD demonstrates a beneficial role in enhancing QoL by ameliorating this syndrome. In addition to improving physical symptoms, TPACD exerted notable effects on emotional functioning, particularly by reducing psychological distress. Research indicates that a significant proportion of patients receiving postoperative adjuvant therapy experience fear of cancer recurrence; approximately 60% experience at least moderate fear, while about 20% report severe fear (Luigjes-Huizer et al., 2022). Hence, the positive impact of TPACD on emotional wellbeing is crucial. Notably, TPACD recipients after the fourth chemotherapy cycle reported fewer financial difficulties than patients in the placebo group, likely due to decreased demand for medications managing CIARs. This study also evaluated alterations in tumor markers. Both groups exhibited a slight upward trend in these markers, although all values remained within the normal range. We speculate that this observation might be attributable to transient factors such as tumor cell lysis following therapy, which can release intracellular tumor antigens, and to chemotherapy-induced inflammatory responses (Goldstein and Mitchell, 2005; Yau et al., 2010). However, as no disease progression was observed, the clinical relevance of this minor increase is considered uncertain. Some studies propose that a transient tumor marker surge within an acceptable range during adjuvant chemotherapy for CRC may indicate a more favorable prognosis (Lehtomäki et al., 2023).

**FIGURE 7:**
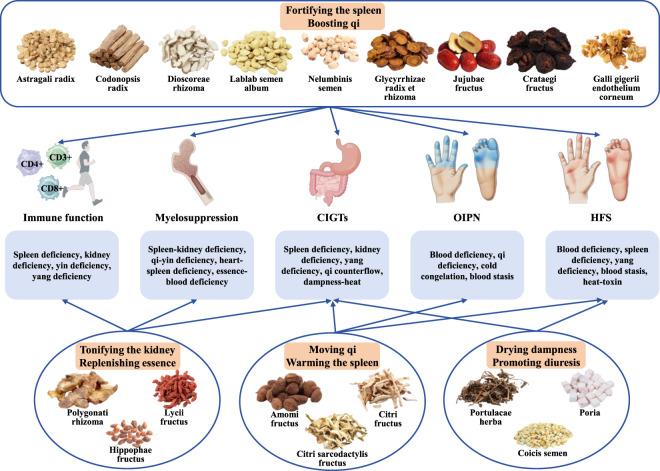
The correspondence between the efficacy of TPACD and TCM syndrome related CIARs.

Based on TCM theory, chemotherapy may further deplete vital Qi, leading to progressive aggravation of spleen-kidney deficiency syndrome as treatment progresses. Owing to the relatively mild potency of materials used in medicines and foods, TPACD combines multiple medicinal materials with synergistic properties to augment its effects. CIGTs are chiefly attributed to spleen deficiency, disruption of qi movement, and the intermingling of dampness and heat. Medicinal materials with corresponding therapeutic effects have demonstrated effectiveness in mitigating these adverse reactions (Zhai et al., 2023). Components within TPACD, including Dioscoreae rhizoma, Nelumbinis semen, and Portulacae herba, may exert such effects. Systemic symptoms, notably fatigue, are correlated with deficiencies of both the spleen and kidney. Medicinal materials with corresponding therapeutic effects have been evidenced to alleviate fatigue induced by chemotherapy (Yang et al., 2023). Specific medicinal materials of TPACD, such as Codonopsis radix and Lycii fructus, are implicated in mediating these beneficial effects. OIPN is associated with qi deficiency, yang deficiency, and blood stasis according to TCM principles. Medicinal materials with properties of supplementing qi, warming yang, and promoting blood circulation have shown benefits in managing OIPN (Han et al., 2024). Medicinal materials within TPACD, including Astragali radix, Jujubae fructus, and Galli gigerii endothelium corneum, are hypothesized to contribute to these effects. It was also observed that TPACD exhibited limited efficacy in addressing myelosuppression, HFS, and pain, and this limitation may stem from the fact that the formulation lacks medicinal materials possessing the specific properties required to target these conditions effectively. For example, managing myelosuppression typically requires medicinal materials that nourish blood and replenish essence (Li et al., 2024). However, such agents frequently possess intestinal-moistening properties that can predispose individuals to diarrhea, thereby justifying their restricted application within TPACD. The management of HFS necessitates medicinal materials that clear heat-toxin and invigorate blood circulation (Deng and Sun, 2018), properties that are seldom found among materials used in medicines and foods. Furthermore, medicinal materials commonly employed for pain alleviation often exhibit mild toxicity (Luo et al., 2020), rendering them similarly unavailable for inclusion under the materials used in medicines and foods classification (Figure 7).

Modern pharmacological studies evaluating the bioactive metabolites identified within TPACD further substantiate these clinical findings. For example, quercetin demonstrates peripheral antiemetic properties (Abolfazli et al., 2024). Polygonatum sibiricum polysaccharides mitigate fatigue through the reduction of oxidative stress (Horng et al., 2014). Formononetin attenuates pain sensitivity associated with OIPN via a neuroprotective mechanism (Mao et al., 2025). Furthermore, wolfiporia cocos polysaccharides have been shown to regulate immune function by increasing peripheral CD4^+^ T cell counts and improving intestinal barrier integrity by enhancing the villus height to crypt depth ratio (Lv et al., 2024).

Unlike previous clinical trials of TCM frequently limited by confounding factors, this investigation implemented rigorous inclusion and exclusion criteria. Although the sample size was modest, it was adequate to meet statistical power requirements. The study’s design, conduct, and reporting complied with the CONSORT Extension for Chinese Herbal Meicine Formulas guidelines (Cheng et al., 2017). For the analysis of TCM syndrome scores and QoL scores, the LMM was applied to adjust for baseline values and examine intervention-time interaction effects.

However, several limitations of this study must be considered. First, baseline characteristics such as surgical stoma status and occupational nature were documented but not incorporated into subgroup analyses, as the existing literature does not support a strong correlation between these factors and the primary outcomes. Second, alleviation of OIPN generally occurs no sooner than 3 months post-anticancer treatment, may progress for up to 6 months, and often persists for extended periods (Pachman et al., 2016). Consequently, the four-cycle chemotherapy observation period in this study was insufficient for evaluating the ultimate efficacy of TPACD on OIPN. Third, although TPACD was associated with a lower grade of CIARs, it did not increase the RDI of CAPOX. Givenits positive effect on the CD4+/CD8+ ratio, the survival benefits indicated in the retrospective analysis may be primarily attributed to the immunomodulatory properties of TPACD rather than the increase in RDI. Furthermore, RDI is an index that reflects both the duration of chemotherapy and the actual dosage of medication. Researchers generally do not deliberately increase the dosage regimen, which is determined by the patient’s overall condition before treatment begins. Fourth, the brief hospitalization period limited the systematic collection and analysis of detailed data on all concurrent medications used during the trial. Comprehensive clinical education prior to enrollment successfully minimized non-essential medication use and strictly restricted the unguided consumption of health products. Nevertheless, despite the limited influence of recorded concomitant medications (primarily nonsteroidal anti-inflammatory drugs), the potential for residual pharmacological confounding cannot be entirely ruled out. Fifth, although rigorous sensory consistency evaluations were conducted prior to the trial to ensure TPACD and placebo were indistinguishable in appearance and taste, formal blinding assessments utilizing quantitative tools, such as Bang’s Blinding Index, were not administered to the participants. Finally, the heterogeneity and individual variation in CIARs precluded clear differentiation between specific adverse events in this study.

## 5 Conclusion

This study demonstrates that TPACD, a formulation composed of materials used in medicines and foods, confers benefits to postoperative CRC patients by attenuating the exacerbation of spleen-kidney deficiency syndrome, alleviating toxicity associated with CAPOX, enhancing QoL, and regulating immune function. Thus, TPACD represents an effective, safe, and well-tolerated complementary therapy for these patients. Because survival data have not yet matured due to the limited follow-up duration, this interim analysis focuses exclusively on toxicity and QoL profiles. The final assessment of long-term survival outcomes will be detailed in a subsequent report upon trial completion.

## Data Availability

The original contributions presented in the study are included in the article/Supplementary Material, further inquiries can be directed to the corresponding authors.
