# Supplementary material for: Efficacy and safety of TiaoPi AnChang decoction to reduce CAPOX-Induced adverse reactions in Chinese patients with colorectal cancer: a randomized, double-blind, placebo-controlled trial
Source: Front Pharmacol. 2026 May 29;17:1707147. doi: 10.3389/fphar.2026.1707147 (PMC13260633; doi:10.3389/fphar.2026.1707147)
Supplement: Supplementary file 1 [file Table1.docx]

Supplementary Material

# SECTION 1

**TPACD preparation**

TPACD consisted of 168 g of medicinal materials, including Astragali radix, Codonopsis radix, Poria, Coicis semen, Dioscoreae rhizoma, Nelumbinis semen, Amomi fructus, Citri sarcodactylis fructus, Citri fructus, Portulacae herba, Lablab semen album, Crataegi fructus, Lycii fructus, Polygonati rhizoma, Hippophae fructus, Galli gigerii endothelium corneum, Glycyrrhizae radix et rhizoma, Jujubae fructus, in a ratio of 15:10:10:10:10:10:6:10:10:10:10:10:10:10:6:10:6:5.

**Quality certificates of the TCM substances**

The authentication, purity assessment, and trace residue analysis (covering sulfur dioxide, pesticides, and microbiological contamination) of the medicinal materials were exclusively conducted by Beijing Meikangtang Pharmaceutical Technology Co., Ltd. (Beijing, China). All tested parameters strictly complied with the guidelines of the Pharmacopoeia of the People’s Republic of China (2020 edition).

**Supplementary Table S0. Quality certificates of the TCM substances.**

| **Drug name (pharmacopoeial)** | **Batch number** | **Area of collection** | **Test report number** |
| --- | --- | --- | --- |
| Astragali radix | 220829003 | Gansu, China | C2208048 |
| Codonopsis radix | 211230002 | Gansu, China | C2112107 |
| Poria | 220216001, | Anhui, China | C2202006 |
| Coicis semen | 220805002 | Fujian, China | C2208009 |
| Dioscoreae rhizoma | 221019003 | Henan, China | C2210035 |
| Nelumbinis semen | 221112003 | Hunan, China | C2211022 |
| Amomi fructus | 211117001 | Guangdong, China | C2111060 |
| Citri sarcodactylis fructus | 210521001 | Guangdong, China | C2105053 |
| Citri fructus | 211114001 | Sichuan, China | C2111051 |
| Portulacae herba | 220304003 | Sichuan, China | C2203010) |
| Lablab semen album | 220801003 | Anhui, China | C2208003 |
| Crataegi fructus | 220304002 | Shandong, China | C2203005 |
| Lycii fructus | 211125001 | Ningxia, China | C2111086 |
| Polygonati rhizoma | 220915002 | Guizhou, China | C2209036 |
| Hippophae fructus | 211015002 | Sichuan, China | C2110035 |
| Galli gigerii endothelium corneum | 220701003 | Henan, China | C2207003 |
| Glycyrrhizae radix et rhizoma | 220917001 | Neimenggu, China | C2209014 |
| Jujubae fructus | 220610003 | Shandong, China | C2206043 |

# SECTION 2 (chemical characterization of TPACD)

**UHPLC-QE-MS/MS**

**Chromatography and mass spectrometry conditions**

Chromatographic separation was performed on an ACQUITY UPLC HSS T3 column (2.1 × 100 mm, 1.8 μm). The column temperature was maintained at 35 ℃. The mobile phase consisted of deionized water containing 0.1% formic acid (A) and acetonitrile containing 0.1% formic acid (B). Gradient elution was conducted at a flow rate of 0.3 mL/min with an injection volume of 10 μL.

Mass spectrometric data were acquired using a Q Exactive Orbitrap high-resolution mass spectrometer operated in Full MS–ddMS² mode, with positive- and negative-ion modes scanned separately. The scan range was m/z 100–1200. The MS¹ and MS² resolutions were set to 70,000 and 17,500, respectively. The spray voltage was 3.2 kV. The capillary temperature and auxiliary gas heater temperature were set to 320 °C and 350 °C, respectively. The sheath gas flow rate was 40 L/min and the auxiliary gas flow rate was 15 L/min. The AGC target was set to 1 × 10⁶, with TopN set to 5. Stepped normalized collision energies (NCE) of 30, 40, and 50 were applied for MS² fragmentation.

A. TIC of TPACD in positive ion mode.

B. TIC of TPACD in negative ion mode.

**Figure S1-1. Total Ion Chromatograms (TICs) of TPACD by UHPLC-QE-MS/MS.**

**Supplementary Table S1-1. The identification information of the metabolites in the TPACD sample** **by UHPLC-QE-MS/MS.**

| **Metabolite name** | **Formula** | **Ms error (ppm)** | **Theoretical mass** | **Observed RT (min)** | **Match score** | **Peak area** |
| --- | --- | --- | --- | --- | --- | --- |
| Citric acid | C6H8O7 | -0.92 | 192.02683 | 1.89 | 91.8 | 13284865103 |
| Stachydrine | C7H13NO2 | 0.74 | 143.09473 | 1.715 | 81.2 | 8920893814 |
| 2-Pyrrolidinecarboxylic acid | C5H9NO2 | 0.93 | 115.06344 | 1.737 | 83.5 | 4161123858 |
| Betaine | C5H11NO2 | 0.58 | 117.07905 | 1.775 | 88.4 | 4041910360 |
| 5-Hydroxymethylfurfural | C6H6O3 | 0.86 | 126.0318 | 15.57 | 85.5 | 1931088541 |
| Sucrose | C12H22O11 | -1.36 | 342.11575 | 1.705 | 93.7 | 1861689147 |
| Liquiritin | C21H22O9 | -0.78 | 418.12605 | 22.543 | 85.6 | 1090552891 |
| L-Leucine | C6H13NO2 | 1.22 | 131.09479 | 4.951 | 83.5 | 979060807.8 |
| Lactose | C12H22O11 | -0.83 | 328.09998 | 1.701 | 83.7 | 897477360 |
| Isoliquiritigenin | C15H12O4 | -0.53 | 256.07342 | 22.566 | 90.5 | 850612582.2 |
| Liguiritigenin-7-O-β-D-apiosyl-4'-O-β-D-glucoside | C26H30O13 | -0.89 | 550.16815 | 22.327 | 88.9 | 746157136.8 |
| Adenosine | C10H13N5O4 | 0.35 | 267.09685 | 11.726 | 89.4 | 624275089.2 |
| Diammonium glycyrrhizinate | C42H62O16 | -0.45 | 822.40342 | 31.012 | 91.6 | 617656321.1 |
| 18 β-Glycyrrhetintic Acid | C30H46O4 | 0.35 | 470.33977 | 31.008 | 86.4 | 509103914.9 |
| Cholic acid | C24H40O5 | -0.69 | 408.28729 | 33.273 | 85.2 | 429724776.9 |
| Epiberberine | C20H17NO4 | -0.33 | 335.11565 | 26.168 | 91.3 | 344883839.6 |
| Citropten | C11H10O4 | 0.59 | 206.05803 | 30.563 | 88.9 | 306146097.9 |
| Calycosin | C16H12O5 | -0.29 | 284.06839 | 26.699 | 90.3 | 304613416.3 |
| Calycosin-7-O-β-D-glucoside | C22H22O10 | -0.21 | 446.1212 | 22.27 | 84.7 | 302127619.8 |
| Ononin | C22H22O9 | -0.05 | 430.12636 | 24.926 | 88 | 290395876.3 |
| Formononetin | C16H12O4 | -0.43 | 268.07344 | 30.968 | 86 | 207727233.2 |
| Manninotriose | C18H32O16 | -0.47 | 504.1688 | 2.678 | 82.4 | 187520739.2 |
| Azelaic acid | C9H16O4 | -0.83 | 188.1047 | 24.208 | 84.4 | 184911428.4 |
| Nicotinic acid | C6H5NO2 | 1.01 | 123.03215 | 3.009 | 80 | 178937402.8 |
| Uridine | C9H12N2O6 | -0.55 | 244.0694 | 6.04 | 93.4 | 174953759.9 |
| Isoliquiritin | C21H22O9 | -0.48 | 418.12618 | 24.857 | 88.1 | 156741853.1 |
| Obacunone | C26H30O7 | 0.32 | 454.1993 | 36.113 | 80.6 | 149900573.9 |
| Lobetyolin | C20H28O8 | -0.56 | 396.17819 | 24.212 | 86.5 | 147107749.1 |
| Cytosine | C4H5N3O | 0.84 | 111.04335 | 3.056 | 76.8 | 146113639.4 |
| p-Coumaric acid | C9H8O3 | 1.2 | 164.04754 | 5.737 | 86.2 | 141860686.2 |
| Rutin | C27H30O16 | -0.55 | 610.15305 | 22.192 | 90.1 | 133647326 |
| Raffinose | C18H32O16 | -0.57 | 504.16875 | 1.698 | 83.5 | 130227351.3 |
| Glabrolide | C30H44O4 | -0.89 | 486.33465 | 28.423 | 74.9 | 107618090.2 |
| Salicylic acid | C7H6O3 | -1.28 | 138.03152 | 24.815 | 87.7 | 95451874.92 |
| Stachyose | C24H42O21 | -0.05 | 666.22183 | 2.577 | 88.8 | 94655641.52 |
| Guanosine | C10H13N5O5 | 0.39 | 283.09178 | 14.409 | 84.6 | 93448142.76 |
| Coptisine chloride | C19H14ClNO4 | -0.47 | 319.08452 | 24.646 | 88.6 | 93015744.66 |
| Narcissoside | C28H32O16 | -0.52 | 578.16351 | 23.09 | 90.5 | 79058245.36 |
| L-Glutamic acid | C5H9NO4 | -0.81 | 147.05304 | 1.6 | 81 | 72824958.53 |
| Cryptochlorogenic acid | C16H18O9 | -0.52 | 354.0949 | 20.048 | 87.8 | 72214033.55 |
| Isoscopoletin | C10H8O4 | 0.49 | 192.04235 | 23.011 | 79.7 | 71178790.91 |
| Epicatechin | C15H14O6 | -0.29 | 290.07895 | 20.9 | 91.6 | 66959292.32 |
| Hesperetin | C16H14O6 | -1.12 | 302.0787 | 23.566 | 85.9 | 64163917.53 |
| α-Cyperone | C15H22O | 0.49 | 218.16717 | 29.035 | 74 | 63330032.8 |
| Palmatine | C21H21NO4 | -0.19 | 351.14699 | 25.877 | 90.4 | 60846221.66 |
| Cytidine | C9H13N3O5 | 0.58 | 243.08566 | 3.044 | 77.8 | 59833556.86 |
| Genistein | C15H10O5 | -0.37 | 270.05272 | 28.659 | 91.2 | 54970153.27 |
| p-Hydroxybenzaldehyde | C7H6O2 | -1.36 | 122.03661 | 21.266 | 73.8 | 54505058.24 |
| Protocatechualdehyde | C7H6O3 | -1.07 | 138.03155 | 19.512 | 73.6 | 51877409.56 |
| Liquiritigenin | C15H12O4 | -0.61 | 256.0734 | 26.283 | 85.4 | 48033478.59 |
| Hyperoside | C21H20O12 | -0.17 | 464.0954 | 22.624 | 90.8 | 46651807.2 |
| Protocatechuic acid | C7H6O4 | -1.08 | 154.02644 | 17.993 | 85.2 | 41791135.56 |
| Daidzein | C15H10O4 | -0.35 | 254.05782 | 25.889 | 86 | 41752551.78 |
| Lonicerin | C27H30O15 | -0.26 | 594.15831 | 22.243 | 73.5 | 39635834.12 |
| Schaftoside | C26H28O14 | 0.19 | 564.14801 | 21.233 | 84.2 | 31975805.02 |
| Isomucronulatol 7-O-glucoside | C23H28O10 | -0.05 | 464.16822 | 26.124 | 88.7 | 31909391.18 |
| Icaritin | C21H20O6 | -0.09 | 368.12595 | 34.487 | 76.2 | 31831950.33 |
| Isorhamnetin-3-O-nehesperidine | C28H32O16 | 0.48 | 624.16934 | 23.086 | 81.5 | 30937892.11 |
| Scoparone | C11H10O4 | 0.59 | 206.05803 | 25.367 | 73.3 | 30043776.88 |
| α-Linolenic acid | C18H30O2 | 0.14 | 278.22462 | 41.787 | 89.4 | 28382460.77 |
| (+)-Magnoflorine | C20H23NO4 | 0.22 | 341.16278 | 21.069 | 76.8 | 25435917.89 |
| Morin | C15H10O7 | -0.82 | 302.04241 | 22.194 | 87.3 | 25100715 |
| Emodin-3-methyl ether/Physcion | C16H12O5 | -0.02 | 284.06847 | 26.303 | 89.2 | 24457058.25 |
| Cantharidin | C10H12O4 | -0.29 | 196.0735 | 21.501 | 71.5 | 24331364.83 |
| Naringin | C27H32O14 | 0.22 | 580.17933 | 23.338 | 83.9 | 23615477.77 |
| Quercetin | C15H10O7 | -0.62 | 302.04246 | 26.746 | 90 | 22657829.64 |
| Quercitrin | C21H20O11 | 0.26 | 448.10068 | 23.54 | 90 | 21771857.02 |
| Quillaic acid | C30H46O5 | 0.53 | 486.33478 | 29.854 | 82 | 21160292.42 |
| Eriocitrin | C27H32O15 | 0.06 | 596.17416 | 22.057 | 84 | 20865571.69 |
| Isomeranzin | C15H16O4 | 0.39 | 260.10496 | 25.186 | 87.5 | 20849276.34 |
| Naringenin | C15H12O5 | -0.02 | 272.06847 | 23.847 | 84 | 20390284.88 |
| Isorhamnetin | C16H12O7 | 0.01 | 316.05831 | 23.088 | 82.7 | 19900314.04 |
| Shikimic acid | C7H10O5 | -2.73 | 174.05235 | 2.535 | 73.3 | 19774444.09 |
| Vicenin II | C27H30O15 | -0.26 | 594.15832 | 20.57 | 84.7 | 19425065.7 |
| Genistin | C21H20O10 | -0.41 | 432.10547 | 22.976 | 84.2 | 18610420.77 |
| Daidzin | C21H20O9 | 0.23 | 416.11083 | 21.509 | 73.7 | 15643427.62 |
| Methylnissolin-3-O-glucoside | C23H26O10 | 0.69 | 462.15292 | 25.749 | 78.1 | 15315149.39 |
| Higenamine | C16H17NO3 | 0.65 | 271.12102 | 19.257 | 79 | 15107573.29 |
| Jatrorrhizine | C20H19NO4 | 0.05 | 337.13143 | 24.269 | 85.7 | 14997235.74 |
| Ferulaldehyde | C10H10O3 | 0.73 | 178.06312 | 21.507 | 74.1 | 14772818 |
| Esculetin | C9H6O4 | -1.11 | 178.02641 | 20.72 | 78.9 | 14761024.94 |
| Kaempferol-3-O-rutinoside | C27H30O15 | -0.05 | 480.16518 | 22.941 | 82.3 | 14284626.14 |
| Naringenin chalcone | C15H12O5 | -0.43 | 272.06836 | 28.515 | 87.2 | 14089867.25 |
| 4-Methoxysalicylic acid | C8H8O4 | -1.23 | 168.04205 | 18.85 | 72.8 | 13223481.69 |
| Glabrone | C20H16O5 | -0.04 | 336.09976 | 37.977 | 86.4 | 12331063.09 |
| 6-Gingerol | C17H26O4 | -0.38 | 294.183 | 34.437 | 82.9 | 12244068.19 |
| Oxypeucedan hydrate | C16H16O6 | -0.02 | 304.09468 | 26.538 | 81.2 | 11956293.07 |
| Tectoridin | C22H22O11 | 0.05 | 462.11623 | 23.776 | 70.8 | 11871255.77 |
| L-Tryptophan | C11H12N2O2 | -0.41 | 204.08979 | 19.065 | 90.9 | 11358195.6 |
| Licochalcone B | C16H14O5 | -0.18 | 286.08407 | 25.794 | 83.3 | 10887354.05 |
| Coumarin | C9H6O2 | -0.45 | 146.03671 | 24.675 | 71.2 | 10747314.75 |
| Typhaneoside | C34H42O20 | -0.02 | 770.22693 | 21.815 | 89.4 | 10245888.75 |
| Astragaloside IV | C41H68O14 | 0.2 | 784.46106 | 30.398 | 78.2 | 10234748.37 |
| Methyl hexadecanoate | C17H34O2 | -0.56 | 316.26094 | 39.418 | 84.9 | 10112420.09 |
| Nuciferine | C19H21NO2 | 0.05 | 295.15724 | 25.089 | 83.3 | 9827142.698 |
| Tectorigenin | C16H12O6 | -0.74 | 300.06317 | 26.276 | 76.5 | 9657072.866 |
| Benzoic acid | C7H6O2 | 1.17 | 122.03692 | 5.728 | 87.9 | 9001313.943 |
| Apigenin 7-O-glucuronide | C21H18O11 | 0.24 | 446.08502 | 23.082 | 87.9 | 6757960.096 |
| Sibiricose A5 | C22H30O14 | -0.53 | 518.16328 | 20.493 | 83.5 | 6382655.126 |
| Medicarpin | C16H14O4 | 0.04 | 270.08922 | 26.739 | 83.7 | 5042737.544 |
| Hydroxygenkwanin | C16H12O6 | -0.88 | 300.06312 | 29.124 | 76.2 | 5041471.501 |
| Rosarin | C20H28O10 | -0.69 | 428.16795 | 23.841 | 75.3 | 4765533.908 |
| Abscisic acid | C15H20O4 | -0.72 | 264.13597 | 26.145 | 74.5 | 4411490.237 |

**UPLC-IM-QTOF-MS/MS**

**Chromatography and mass spectrometry conditions**

Chromatographic separation was performed on an ACQUITY UPLC HSS T3 column (2.1 × 100 mm, 1.8 μm). The column temperature was maintained at 35 ℃. The mobile phase consisted of deionized water containing 0.1% formic acid (A) and acetonitrile containing 0.1% formic acid (B). Gradient elution was conducted as follows: 0–1.5 min, 100% A; 1.5–70 min, linear change from 100% A to 0% A (0% B to 100% B); 70–72 min, maintained at 0% A (100% B). The flow rate was 0.25 mL/min, and the injection volume was 10 μL.

Mass spectrometric data were acquired on a quadrupole time-of-flight (Q-TOF) high-resolution mass spectrometer operated in MS^e^ mode, with positive- and negative-ion modes scanned separately. The scan range was m/z100–1500. The source voltage was set to 2.0 kV, and the cone voltage was 20 V. The ion source temperature and desolvation temperature were maintained at 120 °C and 360 °C, respectively. The cone gas flow and desolvation gas flow were 50 L/h and 600 L/h, respectively. For MS^e^ acquisition, stepped normalized collision energies (NCE) of 20, 35, and 50 V were applied for fragmentation.


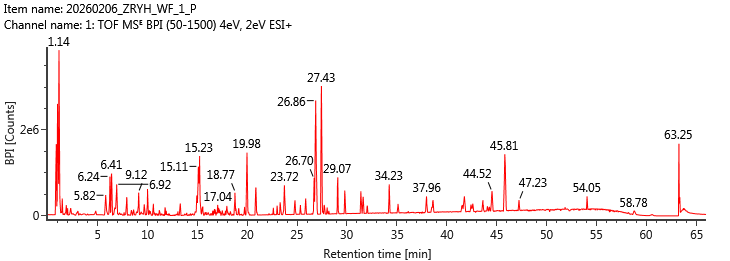


A. TIC of TPACD in positive ion mode.


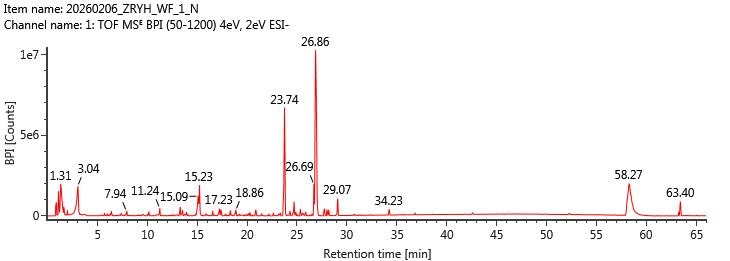


B. TIC of TPACD in negative ion mode.

**Figure S1-2. Total Ion Chromatograms (TICs) of TPACD by UPLC-IM-QTOF-MS/MS.**

**Supplementary Table S1-2. The identification information of the metabolites in the TPACD sample** **by UPLC-IM-QTOF-MS/MS.**

| **Metabolite name** | **Formula** | **Neutral mass (Da)** | **Observed m/z** | **Ms error (ppm)** | **Observed RT (min)** | **Response** |
| --- | --- | --- | --- | --- | --- | --- |
| Glycyrrhizic acid | C42H62O16 | 822.40379 | 821.3974 | 1.1 | 26.71 | 896707 |
| Citric acid | C6H8O7 | 192.027 | 191.0194 | -1.6 | 1.32 | 843133 |
| Isoliquiritin apioside | C26H30O13 | 550.16864 | 549.1614 | 0.1 | 15.1 | 762703 |
| Isoliquiritin | C21H22O9 | 418.12638 | 417.1191 | 0 | 15.22 | 666767 |
| Liquiritigenin | C15H12O4 | 256.07356 | 257.0811 | 1.2 | 15.2 | 253134 |
| Hesperidin | C28H34O15 | 610.18977 | 609.1827 | 0.3 | 17.22 | 236252 |
| Dibutyl phthalate | C16H22O4 | 278.15181 | 301.1413 | 0.8 | 41.75 | 193394 |
| Isorhamnetin-7-O-rhamnoside-3-O-glucoside | C28H32O16 | 624.16903 | 623.1617 | -0.1 | 13.29 | 183476 |
| Z-Nerolidol | C15H26O | 222.19837 | 223.2057 | 0.3 | 44.53 | 175396 |
| 3'-Methoxydaidzin | C16H12O5 | 284.06847 | 285.0757 | -0.3 | 20.87 | 142837 |
| Quinic acid | C7H12O6 | 192.06339 | 191.0554 | -3.7 | 1.14 | 118830 |
| Formononetin | C16H12O4 | 268.07356 | 269.0817 | 3.4 | 18.78 | 117171 |
| Neoisoliquiritin | C21H22O9 | 418.12638 | 417.1192 | 0.3 | 18.87 | 114723 |
| Rutin | C27H30O16 | 610.15338 | 609.1461 | -0.1 | 15.07 | 103598 |
| p-Hydroxybenzyl malic acid | C11H12O6 | 240.06339 | 239.0558 | -1.3 | 11.24 | 99617 |
| Tamarixetin-3-O-β-D-glucoside-7-O-α-L-rhamnoside | C28H32O16 | 624.16903 | 623.1622 | 0.7 | 16.55 | 99222 |
| Scoparone | C11H10O4 | 206.05791 | 207.0653 | 0.7 | 24.79 | 98223 |
| Citropten | C11H10O4 | 206.05791 | 207.0653 | 0.7 | 24.79 | 98223 |
| Codonopsine | C14H21NO4 | 267.14706 | 268.1546 | 1.1 | 7.93 | 94950 |
| Limonin | C26H30O8 | 470.19407 | 471.2013 | 0 | 27.72 | 94917 |
| Calycosin | C16H12O5 | 284.06847 | 285.0761 | 1.1 | 14.94 | 84810 |
| 3'-Methoxy-5'-hydroxyisoflavone-7-O-β-D-glucoside | C22H22O10 | 446.1213 | 447.1287 | 0.3 | 14.94 | 84440 |
| Calycosin-7-O-β-D-glucoside | C22H22O10 | 446.1213 | 447.1287 | 0.3 | 14.94 | 84440 |
| Diosmin | C28H32O15 | 608.17412 | 607.1675 | 1 | 17.13 | 84324 |
| Obacunone | C26H30O7 | 454.19915 | 455.2066 | 0.4 | 32.03 | 69361 |
| 2-Hydroxybutanedioic acid (Malic acid) | C4H6O5 | 134.02152 | 133.0134 | -6 | 1.66 | 67391 |
| Betaine | C5H11NO2 | 117.07898 | 118.0861 | -1.1 | 1.1 | 63239 |
| Valine | C5H11NO2 | 117.07898 | 118.0861 | -1.1 | 1.1 | 63239 |
| Kaempferide-3-O-glucoside | C22H22O11 | 462.11621 | 461.1083 | -1.4 | 15.87 | 57974 |
| Isoliquiritolide (Isoliquiritin lactone) | C30H44O4 | 468.32396 | 469.3313 | 0 | 23.8 | 48112 |
| 3,4,2'-Trihydroxychalcone-4'-O-β-D-glucopyranoside | C21H22O10 | 434.1213 | 433.1143 | 0.7 | 17.28 | 38570 |
| Isoliquiritigenin | C15H12O4 | 256.07356 | 255.0662 | -0.3 | 15.22 | 35836 |
| (6aR,11aR)-10-Hydroxy-3,9-dimethoxypterocarpan | C17H16O5 | 300.09977 | 301.1073 | 0.9 | 19.95 | 33231 |
| Continentalic acid | C30H48O3 | 456.36035 | 455.3532 | 0.2 | 48.13 | 30454 |
| Glycyrol | C30H48O3 | 456.36035 | 455.3532 | 0.2 | 48.13 | 30454 |
| 5,7,4'-Trihydroxyflavone-8-C-α-L-rhamnopyranosyl-(1→2)-β-D-glucopyranoside | C27H30O14 | 578.16356 | 577.1562 | -0.1 | 14.88 | 30145 |
| Apigenol | C15H10O5 | 270.05282 | 269.0456 | 0.1 | 23.16 | 30076 |
| Meconic acid | C18H32O2 | 280.24023 | 279.2328 | -0.6 | 49.85 | 26862 |
| Glucuronolactone | C19H22O3 | 298.15689 | 297.1529 | 10.9 | 37.31 | 26783 |
| Quercetin | C15H10O7 | 302.04265 | 303.0504 | 1.7 | 15.07 | 25460 |
| Glycyrin | C30H44O4 | 468.32396 | 469.3305 | -1.5 | 25.33 | 24571 |
| 1,1-Diethoxy-n-nonane | C13H28O2 | 216.20893 | 239.2005 | 9.7 | 45.21 | 24065 |
| Liensinine | C37H42N2O6 | 610.30429 | 633.2971 | 5.7 | 15.88 | 23987 |
| α-Monolinolenin | C21H38O4 | 354.27701 | 355.2819 | -6.7 | 48.03 | 23502 |
| Phenylpropionic acid | C9H11NO2 | 165.07898 | 166.0867 | 2.6 | 6.92 | 22494 |
| Isorhamnetin | C16H12O7 | 316.0583 | 317.0654 | -0.7 | 16.55 | 22340 |
| d-Catechin | C15H14O6 | 290.07904 | 289.0716 | -0.6 | 12.59 | 22176 |
| Hexadecanoic acid (Palmitic acid) | C16H32O2 | 256.24023 | 255.2323 | -2.4 | 52.29 | 21620 |
| Ethyl myristate | C16H32O2 | 256.24023 | 255.2323 | -2.4 | 52.29 | 21620 |
| Elaidic acid | C18H34O2 | 282.25588 | 281.2485 | -0.2 | 53.23 | 20091 |
| 2'-Hydroxy-3',4'-dimethoxyisoflavan-7-O-β-D-glucoside | C23H28O10 | 464.16825 | 463.1609 | -0.1 | 20.51 | 19974 |
| 18β-Glycyrrhetinic acid | C30H46O4 | 470.33961 | 471.3468 | -0.3 | 26.71 | 19710 |
| 3β,16α-Dihydroxylanosta-7,9(11),24-trien-21-oic acid | C30H46O4 | 470.33961 | 471.3468 | -0.3 | 26.71 | 19710 |
| Naringin | C27H32O14 | 580.17921 | 579.1723 | 0.6 | 16.85 | 19442 |
| Isoquercitrin | C21H20O12 | 464.09548 | 463.0879 | -0.6 | 15.59 | 19296 |
| Liquiritin | C21H22O9 | 418.12638 | 419.1338 | 0.4 | 18.87 | 18907 |
| Allantoin | C4H6N4O3 | 158.04399 | 159.05 | -7.7 | 0.85 | 17903 |
| trans-p-Coumaric acid | C9H8O3 | 164.04734 | 163.0398 | -1.6 | 14.12 | 17660 |
| Vanillic acid-β-D-glucopyranosyl ester | C14H18O9 | 330.09508 | 329.0875 | -1 | 9.3 | 17612 |
| Octadecanoic acid (Stearic acid) | C18H36O2 | 284.27153 | 283.2643 | 0 | 56.81 | 16342 |
| Ethyl palmitate | C18H36O2 | 284.27153 | 283.2643 | 0 | 56.81 | 16342 |
| Fumaric acid | C4H4O4 | 116.01096 | 115.0027 | -8.7 | 1.16 | 16306 |
| Ethyl palmitoleate | C18H34O2 | 282.25588 | 281.2485 | -0.4 | 53.38 | 15591 |
| Palmitoleic acid | C16H30O2 | 254.22458 | 253.2174 | 0.5 | 48.88 | 14931 |
| 6-Methoxy-7-hydroxycoumarin (Scopoletin) | C10H8O4 | 192.04226 | 193.0501 | 3.1 | 15.03 | 14017 |
| 7-Hydroxy-5-methoxycoumarin | C10H8O4 | 192.04226 | 193.0501 | 3.1 | 15.03 | 14017 |
| Neoliquiritin | C21H22O9 | 418.12638 | 417.119 | -0.1 | 19.28 | 13840 |
| Diosmetin | C16H12O6 | 300.06339 | 301.0701 | -1.8 | 17.14 | 13743 |
| 11-Hydroxy-9-tridecenoic acid | C13H24O3 | 228.17254 | 251.1601 | -6.7 | 0.89 | 13181 |
| Kaempferol-3-O-β-D-glucoside (Astragalin) | C21H20O11 | 448.10056 | 447.0937 | 0.9 | 16.97 | 12906 |
| Isopersicarin | C21H20O11 | 448.10056 | 447.0937 | 0.9 | 16.97 | 12906 |
| Quercetin-3-O-α-L-rhamnoside (Quercitrin) | C21H20O11 | 448.10056 | 447.0937 | 0.9 | 16.97 | 12906 |
| Persicarin | C21H20O11 | 448.10056 | 447.0937 | 0.9 | 16.97 | 12906 |
| Quercetin-3-O-β-D-glucopyranoside | C21H20O12 | 464.09548 | 463.0876 | -1.2 | 15.38 | 12845 |
| Quercetin-3-methyl ether | C16H12O7 | 316.0583 | 315.0512 | 0.6 | 24.13 | 12694 |
| L-Norepinephrine | C8H11NO3 | 169.07389 | 192.0653 | 11.2 | 1.21 | 12327 |
| Isorhamnetin-3-O-glucoside | C22H22O12 | 478.11113 | 477.1043 | 1 | 17.11 | 12065 |
| p-Hydroxybenzoic acid | C7H6O3 | 138.03169 | 137.0236 | -5.6 | 16.9 | 11682 |
| o-Hydroxybenzoic acid (Salicylic acid) | C7H6O3 | 138.03169 | 137.0236 | -5.6 | 16.9 | 11682 |
| Proline | C5H9NO2 | 115.06333 | 116.0704 | -1.5 | 0.99 | 11654 |
| Chlorogenic acid | C16H18O9 | 354.09508 | 353.0879 | 0.3 | 10.92 | 11416 |
| (-)-Epicatechin | C15H14O6 | 290.07904 | 289.0717 | -0.1 | 11.02 | 9636 |
| Genistein | C15H12O5 | 272.06847 | 273.0759 | 0.6 | 17.28 | 9455 |
| Pyruvic acid | C3H4O3 | 88.01604 | 87.0077 | -11.7 | 3.03 | 9210 |
| Umbelliferone | C9H6O3 | 162.03169 | 163.0392 | 1.5 | 22.22 | 8812 |
| Gallocatechin | C15H14O7 | 306.07395 | 305.07 | 10.9 | 12.13 | 8454 |
| Epigallocatechin | C15H14O7 | 306.07395 | 305.07 | 10.9 | 12.13 | 8454 |
| Linolenic acid | C18H30O2 | 278.22458 | 277.2171 | -0.7 | 46.84 | 8255 |
| Polycanthine | C15H19NO3 | 261.13649 | 284.1244 | -4.7 | 6.84 | 8028 |
| γ-Tocopherol | C28H48O2 | 416.36543 | 439.3577 | 6.9 | 48.1 | 7877 |
| Dehydroeburicoic acid | C30H48O3 | 456.36035 | 455.3537 | 1.5 | 47.38 | 7652 |
| Ursolic acid | C30H48O3 | 456.36035 | 455.3537 | 1.5 | 47.38 | 7652 |
| 2',7-Dihydroxy-3',4'-dimethoxyisoflavan | C17H18O5 | 302.11542 | 301.1085 | 1.1 | 20.51 | 7628 |
| 7,2'-Dihydroxy-3,4'-dimethoxyisoflavan | C17H18O5 | 302.11542 | 301.1085 | 1.1 | 20.51 | 7628 |
| 8,2'-Dihydroxy-7,4'-dimethoxyisoflavan | C17H18O5 | 302.11542 | 301.1085 | 1.1 | 20.51 | 7628 |
| Furfural | C5H4O2 | 96.02113 | 97.0282 | -2.4 | 1.21 | 7588 |
| 5-Isopentenyloxy-7-methoxycoumarin | C15H16O4 | 260.10486 | 259.0976 | 0.2 | 30.18 | 7574 |
| Crataegoside B | C23H20O10 | 456.10565 | 457.1146 | 3.8 | 41.75 | 7475 |
| Crataegoside C | C23H20O10 | 456.10565 | 457.1146 | 3.8 | 41.75 | 7475 |
| Crataegoside D | C23H20O10 | 456.10565 | 457.1146 | 3.8 | 41.75 | 7475 |
| Corosolic acid | C30H48O4 | 472.35526 | 471.3483 | 0.6 | 38.86 | 7322 |
| 2α-Hydroxyursolic acid | C30H48O4 | 472.35526 | 471.3483 | 0.6 | 38.86 | 7322 |
| 3β,16α-Dihydroxylanosta-8,24-dien-21-oic acid | C30H48O4 | 472.35526 | 471.3483 | 0.6 | 38.86 | 7322 |
| Maslinic acid | C30H48O4 | 472.35526 | 471.3483 | 0.6 | 38.86 | 7322 |
| Riboflavin | C17H20N4O6 | 376.13828 | 377.1447 | -2.3 | 12.48 | 7110 |
| Vitamin B2 | C17H20N4O6 | 376.13828 | 377.1447 | -2.3 | 12.48 | 7110 |
| Protocatechuic acid | C7H6O4 | 154.02661 | 153.0186 | -4.7 | 8.28 | 6871 |
| Gentisic acid | C7H6O4 | 154.02661 | 153.0186 | -4.7 | 8.28 | 6871 |
| Nicotinic acid (Niacin) | C6H5NO2 | 123.03203 | 124.0393 | -0.3 | 2.4 | 6857 |
| Ambrettolide | C16H28O2 | 252.20893 | 275.2009 | 9.9 | 23.31 | 6431 |
| 9-Hydroxy-10,12-pentadecadienoic acid | C15H26O3 | 254.18819 | 277.1797 | 8.2 | 40.25 | 6344 |
| n-Pentadecanal | C15H30O | 226.22967 | 249.2217 | 11.3 | 27.82 | 5482 |
| Sorbitol | C6H14O6 | 182.07904 | 181.0714 | -1.9 | 1.08 | 5236 |
| Aspartic acid | C4H7NO4 | 133.03751 | 132.0296 | -4.4 | 1.03 | 4992 |
| Kaempferol-7-O-α-L-rhamnoside | C21H20O10 | 432.10565 | 433.1136 | 1.6 | 15.93 | 4916 |
| Genistein-7-O-β-D-glucoside (Genistin) | C21H20O10 | 432.10565 | 433.1136 | 1.6 | 15.93 | 4916 |
| Agroastragaloside III | C51H82O21 | 1030.53486 | 1053.518 | -5.8 | 26.86 | 4718 |
| Folinic acid | C20H23N7O7 | 473.1659 | 474.1732 | 0 | 9.32 | 4665 |
| 6-Methyl-5-hepten-2-one | C8H14O | 126.10447 | 125.0966 | -4.5 | 17.38 | 4660 |
| Leucine | C6H13NO2 | 131.09463 | 132.1021 | 1.8 | 3.79 | 4597 |
| Poricoic acid B | C30H44O5 | 484.31887 | 485.3256 | -1.2 | 22.19 | 4557 |
| (+)-Syringaresinol | C22H26O8 | 418.16277 | 419.1679 | -5.1 | 17.85 | 4541 |
| Tamarixetin-3-O-β-D-glucopyranoside | C22H22O12 | 478.11113 | 477.104 | 0.4 | 15.93 | 4498 |
| Agroastragaloside IV | C49H80O20 | 988.52429 | 987.5207 | 3.7 | 27.9 | 4413 |
| Oleracein A | C24H25NO11 | 503.14276 | 502.1351 | -0.8 | 14.86 | 4402 |
| Glycybenzofuran | C21H22O5 | 354.14672 | 353.1394 | 0 | 31.65 | 4308 |
| Soyasaponin βg | C47H74O17 | 910.4926 | 933.4776 | -4.5 | 17.96 | 4292 |
| Acetylastragaloside I | C47H74O17 | 910.4926 | 933.4776 | -4.5 | 17.96 | 4292 |
| Kingianoside C | C45H72O20 | 932.46169 | 933.4776 | 9.3 | 17.96 | 4292 |
| Geranylacetone | C13H22O | 194.16707 | 195.1749 | 2.9 | 39.24 | 4211 |
| 7-Methoxycoumarin (Herniarin) | C10H8O3 | 176.04734 | 177.0547 | 0.5 | 15.15 | 4141 |
| Diethyl phthalate | C12H14O4 | 222.08921 | 221.0816 | -1.7 | 25.8 | 4132 |
| Vitamin K1 (Phylloquinone) | C31H46O2 | 450.34978 | 473.3437 | 10 | 44.29 | 4125 |
| 4-Hydroxymethylfurfural | C6H6O3 | 126.03169 | 127.0391 | 1.1 | 1.16 | 4109 |
| Aviprin | C16H16O6 | 304.09469 | 305.1018 | -0.4 | 20.51 | 4034 |
| cis-head-to-head-3,3',4,4'-Citropten dimer | C22H20O8 | 412.11582 | 413.1233 | 0.6 | 17.22 | 4022 |
| cis-head-to-tail-3,3',4,4'-Citropten dimer | C22H20O8 | 412.11582 | 413.1233 | 0.6 | 17.22 | 4022 |
| Licochalcone B | C16H14O5 | 286.08412 | 285.077 | 0.5 | 19.74 | 3982 |
| Astragaline A | C10H11NO4 | 209.06881 | 210.0762 | 0.5 | 1.2 | 3934 |
| 5-Methoxyfurfural | C6H6O3 | 126.03169 | 127.0392 | 2.1 | 7.36 | 3729 |
| Ascorbic acid | C6H8O6 | 176.03209 | 177.0389 | -2.4 | 2.84 | 3706 |
| Vitamin C | C6H8O6 | 176.03209 | 177.0389 | -2.4 | 2.84 | 3706 |
| n-Tridecanal | C13H26O | 198.19837 | 221.1901 | 11.4 | 41.09 | 3688 |
| Isovitexin | C21H20O10 | 432.10565 | 433.1135 | 1.4 | 15.08 | 3630 |
| Vitexin | C21H20O10 | 432.10565 | 433.1135 | 1.4 | 15.08 | 3630 |
| Isoleucine | C6H13NO2 | 131.09463 | 132.1021 | 1.8 | 4.09 | 3593 |
| (+)-Syringaresinol-O-β-D-glucopyranoside | C28H36O13 | 580.21559 | 579.209 | 1.2 | 16.65 | 3518 |
| Isoferulic acid | C10H10O4 | 194.05791 | 193.0503 | -1.5 | 11.85 | 3485 |
| Coumarin | C9H6O2 | 146.03678 | 147.0444 | 2.2 | 14.12 | 3452 |
| Oleracein C | C30H35NO16 | 665.19558 | 664.1891 | 1.2 | 13.41 | 3450 |
| 8,3'-Dihydroxy-7,4'-dimethoxyisoflavone | C17H14O6 | 314.07904 | 315.0871 | 2.5 | 21.43 | 3429 |
| Kaempferol | C15H10O6 | 286.04774 | 287.0547 | -1.3 | 16.29 | 3402 |
| Luteolin | C15H10O6 | 286.04774 | 287.0547 | -1.3 | 16.29 | 3402 |
| Deoxyglycyrol | C30H46O3 | 454.3447 | 453.3378 | 0.9 | 51.48 | 3334 |
| Nuciferine | C19H21NO2 | 295.15723 | 296.1643 | -0.7 | 18.7 | 3321 |
| 3-Methoxy-4-hydroxybenzoic acid (Vanillic acid) | C8H8O4 | 168.04226 | 167.0347 | -2 | 7.53 | 3291 |
| Vitamin Eβ (β-Tocopherol) | C28H48O2 | 416.36543 | 439.3569 | 5.2 | 27.65 | 3284 |
| Santin | C18H16O7 | 344.0896 | 345.0972 | 1 | 17.22 | 3224 |
| 9,10-Dimethoxypterocarpan-3-O-β-D-glucoside | C23H26O10 | 462.1526 | 463.1604 | 1 | 19.95 | 3205 |
| Phenylacetic acid | C8H8O2 | 136.05243 | 137.0596 | -0.7 | 13.52 | 3203 |
| Benzoic acid | C7H6O2 | 122.03678 | 123.0441 | 0.2 | 4.58 | 3096 |
| 5-Hydroxymethylfurfural (5-HMF) | C6H6O3 | 126.03169 | 127.0391 | 0.6 | 8.56 | 3051 |
| Oleracein B | C25H27NO12 | 533.15333 | 532.1463 | 0.5 | 15.35 | 3032 |
| Nicotinamide | C6H6N2O | 122.04801 | 123.055 | -2.2 | 3.08 | 3028 |

# SECTION 3

**Supplementary Table S2. Primary outcome: Incidence of overall CIARs (PPS), n(%).** The Tables regarding CIARs in the main text employed the rank-sum test. Here, the differences in CIARs of each grade are presented, using chi-square test or Fisher’s exact test.

|  | **Placebo Group**  **(N = 53)** | **TPACD Group**  **(N = 49)** | ***P*** |
| --- | --- | --- | --- |
|  |  |  | **0.009** |
| 1 grade | 18 (35.29) | 28 (62.22) | 0.008 |
| 2 grade | 28 (54.90) | 15 (33.33) | 0.034 |
| 3 grade | 5 (9.80) | 2 (4.44) | 0.539 |

**Supplementary Table S3. Primary outcome: Incidence of CIGTs (PPS), n(%).** The Tables regarding CIGTs in the main text employed the rank-sum test. Here, the differences in CIGTs of each grade are presented, using chi-square test or Fisher’s exact test.

|  | **Placebo Group**  **(N = 53)** | **TPACD Group**  **(N = 49)** | ***P*** |
| --- | --- | --- | --- |
| **Overall CIGTs** |  |  |  |
| 1 grade | 28 (54.90) | 42 (93.33) | <0.001 |
| 2 grade | 19 (37.25) | 3 (6.67) | <0.001 |
| 3 grade | 4 (7.84) | 0 (0.00) | 0.159 |
| **Loss of appetite** |  |  |  |
| 0 | 0 (0.00) | 7 (15.56) | 0.011 |
| 1 grade | 38 (74.51) | 37 (82.22) | 0.362 |
| 2 grade | 11 (21.57) | 1 (2.22) | 0.004 |
| 3 grade | 2 (3.92) | 0 (0.00) | 0.497 |
| **Nausea** |  |  |  |
| 0 | 1 (1.96) | 12 (26.67) | <0.001 |
| 1 grade | 37 (72.55) | 32 (71.11) | 0.876 |
| 2 grade | 10 (19.61) | 1 (2.22) | 0.008 |
| 3 grade | 3 (5.88) | 0 (0.00) | 0.287 |
| **Vomiting** |  |  |  |
| 0 | 11 (21.57) | 38 (84.44) | <0.001 |
| 1 grade | 30 (58.82) | 6 (13.33) | <0.001 |
| 2 grade | 9 (17.65) | 1 (2.22) | 0.033 |
| 3 grade | 1 (1.96) | 0 (0.00) | 1 |
| **Diarrhea** |  |  |  |
| 0 | 22 (43.14) | 32 (71.11) | 0.006 |
| 1 grade | 25 (49.02) | 12 (26.67) | 0.025 |
| 2 grade | 4 (7.84) | 1 (2.22) | 0.437 |
| **Constipation** |  |  |  |
| 0 | 40 (78.43) | 41 (91.11) | 0.088 |
| 1 grade | 9 (17.65) | 4 (8.89) | 0.211 |
| 2 grade | 2 (3.92) | 0 (0.00) | 0.497 |
| **Oral mucositis** |  |  |  |
| 0 | 35 (68.63) | 33 (73.33) | 0.615 |

**Supplementary Table S4. Primary outcome: Incidence of myelosuppression (PPS), n(%).** The Tables regarding myelosuppression in the main text employed the rank-sum test. Here, the differences in myelosuppression of each grade are presented, using chi-square test or Fisher’s exact test.

|  | **Placebo Group**  **(N = 53)** | **TPACD Group**  **(N = 49)** | ***P*** |
| --- | --- | --- | --- |
| **Overall myelosuppression** |  |  |  |
| 0 | 17 (33.33) | 19 (42.22) | 0.369 |
| 1 grade | 20 (39.22) | 15 (33.33) | 0.55 |
| 2 grade | 13 (25.49) | 9 (20.00) | 0.523 |
| 3 grade | 1 (1.96) | 2 (4.44) | 0.912 |
| **Leukopenia** |  |  |  |
| 0 | 37 (72.55) | 36 (80.00) | 0.393 |
| 1 grade | 8 (15.69) | 6 (13.33) | 0.744 |
| 2 grade | 6 (11.76) | 3 (6.67) | 0.614 |
| **Neutropenia** |  |  |  |
| 0 | 33 (64.71) | 34 (75.56) | 0.248 |
| 1 grade | 7 (13.73) | 7 (15.56) | 0.8 |
| 2 grade | 10 (19.61) | 4 (8.89) | 0.138 |
| 3 grade | 1 (1.96) | 0 (0.00) | 1 |
| **Thrombocytopenia** |  |  |  |
| 0 | 38 (74.51) | 32 (71.11) | 0.708 |
| 1 grade | 11 (21.57) | 8 (17.78) | 0.642 |
| 2 grade | 2 (3.92) | 3 (6.67) | 0.886 |
| 3 grade | 0 (0.00) | 2 (4.44) | 0.217 |
| **Anemia** |  |  |  |
| 0 | 37 (72.55) | 33 (73.33) | 0.931 |
| 1 grade | 11 (21.57) | 7 (15.56) | 0.451 |
| 2 grade | 3 (5.88) | 5 (11.11) | 0.579 |

**Supplementary Table S5. Incidence of** **CIPN and HFS (PPS), n(%).** The Tables regarding CIPN and HFS in the main text employed the rank-sum test. Here, the differences in CIPN and HFS of each grade are presented, using chi-square test or Fisher’s exact test.

|  | **Placebo Group(N = 53)** | **TPACD Group(N = 49)** | ***P*** |
| --- | --- | --- | --- |
| **OIPN** |  |  |  |
| 0 | 6 (11.76) | 19 (42.22) | <0.001 |
| 1 grade | 39 (76.47) | 22 (48.89) | 0.005 |
| 2 grade | 5 (9.80) | 4 (8.89) | 1.000 |
| 3 grade | 1 (1.96) | 0 (0.00) | 1.000 |
| **HFS** |  |  |  |
| 0 | 29 (56.86) | 34 (75.56) | 0.035 |
| 1 grade | 21 (41.18) | 11 (24.44) | 0.083 |
| 2 rade | 1 (1.96) | 0 (0.00) | 1.000 |

**Supplementary Table S6. TCM syndrome score and EORTC QLQ-C30 score across timepoints (PPS), Mean ± SD.** The drawing data of Figure 3 and Figure 4 in the main text.

|  |  | **Total** | **Placebo Group(N = 53)** | **TPACD Group(N = 49)** |
| --- | --- | --- | --- | --- |
| **TCM syndrome score** | V0 | 4.78 ± 1.30 | 4.53 ± 0.82 | 5.06 ± 1.64 |
|  | V1 | 5.07 ± 1.81 | 5.77 ± 1.48 | 4.31 ± 1.84 |
|  | V2 | 6.18 ± 2.08 | 7.06 ± 1.59 | 5.20 ± 2.13 |
|  | V3 | 6.60 ± 1.95 | 7.68 ± 1.75 | 5.40 ± 1.37 |
|  | V4 | 7.10 ± 2.11 | 8.43 ± 1.67 | 5.64 ± 1.48 |
| **Physical functioning score** | V0 | 87.45 ± 8.82 | 87.80 ± 9.13 | 87.07 ± 8.54 |
|  | V2 | 87.49 ± 8.62 | 86.54 ± 8.99 | 88.55 ± 8.15 |
|  | V4 | 86.38 ± 8.46 | 84.90 ± 9.11 | 88.00 ± 7.47 |
| **Role functioning score** | V0 | 88.56 ± 14.07 | 89.62 ±13.17 | 87.41 ±15.03 |
|  | V2 | 85.05 ± 16.40 | 83.66±16.83 | 86.59 ± 15.96 |
|  | V4 | 81.38 ± 15.62 | 79.93 ±16.31 | 82.96±14.86 |
| **Emotional functional score** | V0 | 92.24 ± 9.03 | 91.82 ± 9.02 | 92.69 ± 9.10 |
|  | V2 | 90.29 ± 9.52 | 88.73 ± 9.98 | 92.03 ± 8.78 |
|  | V4 | 89.89 ± 9.61 | 87.76 ± 9.18 | 92.22 ± 9.63 |
| **cognitive functional score** | V0 | 93.46 ± 10.79 | 93.71 ± 9.93 | 93.20 ± 11.74 |
|  | V2 | 91.75 ± 11.82 | 92.48 ± 11.21 | 90.94 ± 12.52 |
|  | V4 | 89.72 ± 13.41 | 87.76 ± 14.34 | 91.85 ± 12.11 |
| **Social functional score** | V0 | 91.67 ± 11.19 | 92.14 ± 11.13 | 91.16 ± 11.34 |
|  | V2 | 88.66 ± 13.08 | 87.58 ± 13.68 | 89.86 ± 12.41 |
|  | V4 | 85.82 ± 13.38 | 82.65 ± 14.01 | 89.26 ± 11.87 |
| **Global QOL score** | V0 | 80.07 ± 8.94 | 80.03 ± 8.55 | 80.10 ± 9.44 |
|  | V2 | 74.91 ± 10.59 | 72.22 ± 9.81 | 77.90 ± 10.72 |
|  | V4 | 70.92 ± 10.98 | 65.31 ± 9.37 | 77.04 ± 9.26 |
| **Fatigue score** | V0 | 22.33 ± 11.22 | 21.80 ± 10.67 | 22.90 ± 11.87 |
|  | V2 | 24.74 ± 12.06 | 28.10 ± 12.24 | 21.01 ± 10.79 |
|  | V4 | 29.79 ± 11.31 | 33.79 ± 11.33 | 25.43 ± 9.66 |
| **Nausea and vomiting score** | V0 | 0.00 ± 0.00 | 0.00 ± 0.00 | 0.00 ± 0.00 |
|  | V2 | 12.54 ± 15.78 | 18.63 ± 16.55 | 5.80 ± 11.77 |
|  | V4 | 16.31 ± 20.30 | 25.51 ± 23.35 | 6.30 ± 8.91 |
| **Pain score** | V0 | 3.92 ± 10.53 | 3.46 ± 11.49 | 4.42 ± 9.49 |
|  | V2 | 8.08 ± 14.85 | 9.80 ± 16.73 | 6.16 ± 12.35 |
|  | V4 | 11.17 ± 15.34 | 13.61 ± 16.20 | 8.52 ± 14.04 |
| **Dyspnoea score** | V0 | 3.59 ± 10.39 | 4.40 ± 11.39 | 2.72 ± 9.22 |
|  | V2 | 2.41 ± 8.67 | 3.27 ± 10.01 | 1.45 ± 6.87 |
|  | V4 | 3.55 ± 11.43 | 4.76 ± 13.61 | 2.22 ± 8.41 |
| **Insomnia score** | V0 | 7.84 ± 15.68 | 6.92 ± 13.65 | 8.84 ± 17.71 |
|  | V2 | 8.93 ± 17.69 | 7.84 ± 17.11 | 10.14 ± 18.42 |
|  | V4 | 10.99 ± 18.54 | 11.56 ± 18.70 | 10.37 ± 18.56 |
| **Appetite score** | V0 | 4.25 ± 11.17 | 3.77 ± 10.66 | 4.76 ± 11.79 |
|  | V2 | 24.05 ± 21.91 | 28.76 ± 24.06 | 18.84 ± 18.12 |
|  | V4 | 29.79 ± 21.01 | 36.73 ± 22.82 | 22.22 ± 15.89 |
| **constipation** | V0 | 4.62 ± 12.50 | 5.66 ± 14.23 | 3.47 ± 10.29 |
|  | V2 | 3.47 ± 10.24 | 4.58 ± 11.58 | 2.22 ± 8.41 |
|  | V4 | 3.94 ± 11.89 | 6.12 ± 14.71 | 1.52 ± 7.02 |
| **Diarrhoea** | V0 | 6.27 ± 13.09 | 6.29 ± 13.17 | 6.25 ± 13.15 |
|  | V2 | 7.64 ± 14.89 | 9.15 ± 15.02 | 5.93 ± 14.72 |
|  | V4 | 9.32 ± 17.28 | 13.61 ± 20.32 | 4.55 ± 11.57 |
| **Financial difficulties** | V0 | 9.15 ± 15.67 | 9.43 ± 16.51 | 8.84 ± 14.87 |
|  | V2 | 9.28 ± 15.77 | 10.46 ± 16.98 | 7.97 ± 14.38 |
|  | V4 | 11.70 ± 16.73 | 13.61 ± 17.90 | 9.63 ± 15.28 |

**Supplementary Table S7. The differences in peripheral blood immune cell subsets between the two groups (PPS), Mean ± SD.**

|  | **(%)** | **CD3+** | **CD4+** | **CD8+** | **CD4+/CD8+** | **NK** | **CD19+** |
| --- | --- | --- | --- | --- | --- | --- | --- |
| **V0** | Placebo Group | 67.46±9.91 | 38.44±9.56 | 25.17±8.46 | 1.72±0.79 | 20.09 ± 10.70 | 10.85 ± 4.45 |
|  | TPACD Group | 69.85±11.17 | 37.73±7.30 | 27.40±10.49 | 1.56±0.59 | 19.13 ± 9.90 | 9.45 ± 4.20 |
|  | t | -1.17 | 0.43 | -1.208 | 1.183 | 0.47 | 1.58 |
|  | *P* | 0.245 | 0.668 | 0.23 | 0.239 | 0.639 | 0.117 |
| **V2** | Placebo Group | 70.40±11.16 | 40.77±9.58 | 25.98±9.52 | 1.81±0.85 | 19.14 ± 11.66 | 8.97 ± 3.28 |
|  | TPACD Group | 74.27±8.75 | 41.58±8.31 | 27.55±11.89 | 1.77±0.77 | 15.84 ± 7.55 | 8.06 ± 4.58 |
|  | t | -1.723 | -0.401 | -0.655 | 0.225 | 1.47 | 1.00 |
|  | *P* | 0.089 | 0.689 | 0.515 | 0.823 | 0.146 | 0.323 |
| **V4** | Placebo Group | 71.93±9.99 | 42.73±8.76 | 25.60±8.52 | 1.91±0.94 | 17.99±10.18 | 8.70 ± 4.42 |
|  | TPACD Group | 73.56±8.32 | 47.68±7.07 | 20.68±8.28 | 2.64±1.04 | 18.37 ± 8.09 | 7.49 ± 3.21 |
|  | t | -0.778 | -2.714 | 2.584 | -3.235 | -0.18 | 1.34 |
|  | *P* | 0.439 | 0.008 | 0.012 | 0.002 | 0.858 | 0.183 |

**Supplementary Table S8. The differences in tumor markers between the two groups (PPS), Median (Lower quartile, Upper quartile).**

|  |  | **CEA, M (Q₁, Q₃)** | **CA199, M (Q₁, Q₃)** |
| --- | --- | --- | --- |
| **V0** | Placebo Group | 1.83(1.32, 2.80) | 9.87(6.54, 16.95) |
|  | TPACD Group | 1.58(1.03, 2.56) | 9.84(5.53, 15.18) |
|  | Z | -1.318 | -0.632 |
|  | *P* | 0.188 | 0.527 |
| **V2** | Placebo Group | 2.70(1.95, 3.96) | 14.28(8.60, 22.28) |
|  | TPACD Group | 2.40(1.91, 3.34) | 10.75(6.48, 17.84) |
|  | Z | -1.134 | -1.036 |
|  | *P* | 0.257 | 0.3 |
| **V4** | Placebo Group | 3.14(1.98, 4.11) | 14.12(9.94, 23.15) |
|  | TPACD Group | 2.93(2.28, 3.82) | 16.65(7.93, 25.51) |
|  | Z | -0.099 | -0.153 |
|  | *P* | 0.921 | 0.879 |

**Supplementary Table S9. Changes in tumor markers across timepoints (PPS), Median (Lower quartile, Upper quartile).**

|  |  | **V0** | **V4** | **Z** | **P** |
| --- | --- | --- | --- | --- | --- |
| **Placebo Group** | CEA, M (Q₁, Q₃) | 1.83(1.32, 2.80) | 3.14(1.98, 4.11) | -3.475 | 0.001 |
|  | CA199, M (Q₁, Q₃) | 9.87(6.54, 16.95) | 14.12(9.94, 23.15) | -2.466 | 0.014 |
| **TPACD Group** | CEA, M (Q₁, Q₃) | 1.58(1.03, 2.56) | 2.93(2.28, 3.82) | -4.665 | ＜0.001 |
|  | CA199, M (Q₁, Q₃) | 9.84(5.53, 15.18) | 16.65(7.93, 25.51) | -2.537 | 0.011 |

**Supplementary Table S10. Incidence of adverse event (PPS), n(%)..**

|  | **Placebo Group**  **(N = 53)** | **TPACD Group**  **(N = 49)** | ***P*** |
| --- | --- | --- | --- |
| Renal injury | 0 | 0 | - |
| Hepatic injury |  |  | 0.380 |
| 0 | 27 (50.94) | 21 (42.86) |  |
| 1 grade | 24 (45.28) | 25 (51.02) |  |
| 2 grade | 2 (3.77) | 3 (6.12) |  |
